# Concomitant pyroptotic and apoptotic cell death triggered in macrophages infected by Zika virus

**DOI:** 10.1371/journal.pone.0257408

**Published:** 2022-04-21

**Authors:** Chunxia Wen, Yufeng Yu, Chengfeng Gao, Xian Qi, Carol J. Cardona, Zheng Xing

**Affiliations:** 1 Jiangsu Provincial Key Laboratory of Medicine, Medical School, Nanjing University, Nanjing, China; 2 Shanxi Provincial Key Laboratory for Functional Proteins, School of Basic Medical Sciences, Shanxi Medical University, Taiyuan, China; 3 Jiangsu Provincial Center for Disease Control and Prevention, Nanjing, China; 4 Department of Veterinary Biomedical Sciences, College of Veterinary Medicine, University of Minnesota at Twin Cities, Saint Paul, Minnesota, United States of America; University of the Pacific, UNITED STATES

## Abstract

Zika virus (ZIKV) is a positive-sense RNA flavivirus and can cause serious neurological disorders including microcephaly in infected fetuses. As a mosquito-borne arbovirus, it enters the bloodstream and replicates in various organs. During pregnancy, it can be transmitted from the blood of the viremic mother to the fetus by crossing the placental barrier. Monocytes and macrophages are considered the earliest blood cell types to be infected by ZIKV. As a first line defense, these cells are crucial components in innate immunity and host responses and may impact viral pathogenesis in humans. Previous studies have shown that ZIKV infection can activate inflammasomes and induce proinflammatory cytokines in monocytes. In this report, we showed that ZIKV could infect and induce cell death in human and murine macrophages. In addition to the presence of cleaved caspase-3, indicating that apoptosis was involved, we identified the cleaved caspase-1 and gasdermin D (GSDMD) as well as increased secretion of IL-1β and IL-18. This suggests that the inflammasome was activated and that may lead to pyroptosis in infected macrophages. The pyroptosis was NLRP3-dependent and could be suppressed in the macrophages treated with shRNA to target and knockdown caspase-1. It was also be inhibited by an inhibitor for caspase-1, indicating that the pyroptosis was triggered via a canonical approach. Our findings in this study demonstrate a concomitant occurrence of apoptosis and pyroptosis in ZIKV-infected macrophages, with two mechanisms involved in the cell death, which may have potentially significant impacts on viral pathogenesis in humans.

## Introduction

Zika virus (ZIKV) is a member of *Flaviviridae* family, which includes a large group of viruses that cause West Nile encephalitis, Dengue Fever, Japanese encephalitis, Tick-borne encephalitis and other important human diseases [1]. ZIKV infection is usually self-limited, and most cases are either asymptomatic or have mild symptoms such as fever, rash, conjunctivitis and malaise. ZIKV has been associated with Guillain-Barre syndrome and other mild neurological symptoms in some adults [2]. The virus caught the world’s attention when it was linked to congenital infections, leading to spontaneous abortions and severe neonatal birth defects including microcephaly when a severe outbreak occurred in South America in late 2015 and into 2016 [3].

Innate immunity plays a critical role in the early phase of viral infections and host defense, in which monocytes and macrophages, originating from bone marrow myeloid progenitor cells are key players [4]. Once an infection occurs, monocytes activate their phagocytic function and release a variety of cytokines and chemokines, which will further promote their activation and differentiation [5,6]. Monocytes can become macrophages when they egress from the bloodstream and invade tissues and organs via chemotaxis. In addition to cytokine and chemokine release, monocytes/macrophages recruit lymphocytes and activate adaptive immunity through antigen presentation [7] and help clear viral infection in the host. On the other hand, monocytes infected with viruses are possible Trojan horses under circumstances that lead to virus spread and dissemination within the host. More importantly, this mechanism can bring viruses into immune privileged tissues and organs such as the placenta, testes, and brain when monocytes migrate across protective blood barriers [8]. Indeed several studies show that monocytes facilitate virus dissemination and transmigration into the brain by traversing the blood-brain barrier (BBB) [9,10]. Additionally, they can serve as virus reservoirs or as niches for viral persistence in connection with chronic post arbovirus infections [11].

Despite the fact that viremia in Zika patients is common, knowledge about the exact target blood cells and their responses in the blood during ZIKV infection remains limited. Blood CD14+ monocytes appear to be the maincells for ZIKV infection, which leads to differential immunomodulatory response or M2-skewed immunosuppression during pregnancy [12]. A recent report shows that monocytes express adhesion molecules and have abilities to attach onto vessel walls and transmigrate across endothelia, which promotes ZIKV dissemination to neural cells [13]. Several studies have shown that ZIKV infection can activate the NLRP3 inflammasome, which results in the secretion of pro-inflammatory cytokines [14–16]. These findings make it complicated to assess the role of monocytes in ZIKV infection, and in particular in viral pathogenesis in an infection during pregnancy. However, no study has shown what the fate is for the infected monocytes [14–17], while placental macrophages appear to be resistant to cell death during ZIKV infection [18].

Host cells can react by activating various innate defenses in response to viral infections. In addition to antiviral or pro-inflammatory cytokines and chemokines, cells can trigger programed cell death with complex outcomes, which may eliminate infected cells and clear virus replicative niche [19]. Apopotosis and pyroptosis are caspase-dependent cell death, but necroptosis is activated relying on the activation of phosphorylated receptor interacting serine/threonine-protein kinase (RIPK) [20–24]. In this report, we showed the evidence that a productive ZIKV infection led to cell death in both human and murine macrophages. Our data indicated that infected macrophages died of apoptosis and pyroptosis with the presence of cleaved caspase-1 and caspase-3 and processed GSDMD. The pyroptosis in human and murine macrophages was dependent on the NLRP3 inflammasome activation induced by ZIKV infection. Although previous studies have indicated the significance of inflammasomes in proinflammatory cytokine responses [14–17] and inhibition of the cGAS-mediated interferon signaling in ZIKV monocytes [14], concomitant apoptosis and pyroptosis may benefit the host by removing a shelter for the virus and preventing viral spreading and disseminating, which could be of significance to viral pathogenesis in human ZIKV infections.

## Materials and methods

### Cell lines and cultures

THP-1 cells and human embryonic kidney cells (HEK293T) were purchased from the Cell Bank of Chinese Academy Sciences (Shanghai, China). RAW264.7 cells were purchased from the American Type Culture Collection (ATCC). RAW264.7 and HEK293T cells were cultured in Dulbecco’s modified Eagle’s medium (DMEM) (Gibco, Grand Island, NY) supplemented with 10% fetal bovine serum (FBS, ExCell Bio., China), 100 U/ml penicillin, and 100 μg/ml streptomycin sulphate (Beyotime Biotechnology, China). Cells were cultured in an incubator at 37°C with a humidified atmosphere of 5% CO_2_.

### Reagents and antibodies

ZIKV-E antibody (#B1845) was purchased from Beijing Biodragon Immunotech (Beijing, China). Antibodies for pro-caspase-1 antibody (#ab179515), phospho-MLKL (#ab187091), phospho-RIPK3 (#209384) and GSDMD (#ab210070) were purchased from Abcam (Cambridge, MA). Another GSDMD antibody (#A10164) was purchased from ABclonal. Antibodies for NLRP3 (#D4D8T), pro-caspase-3 (#9555S), cleaved caspase-3 (#9664S, #9661), pro-PARP (#9532S), cleaved-PARP (#9541S), and phospho-RIPK1 (#44590S) were purchased from Cell Signalling Technology (Beverly, MA). We also purchased antibodies for cleaved caspase-1 (#AF4022) from Affinity Biosciences (Taizhou, China) and GAPDH and β-actin from Proteintech (Wuhan, China). An MTT Assay Kit was purchased from SunShineBio (Nanjing, China). ELISA kits for mouse interleukin-1β, mouse interleukin-18, human interleukin-1β, and human interleukin-18 were purchased from BOSTER (Wuhan, China). Compounds VX765, ZVAD-FMK, GSK’872, Nec-1s, and Phorbol-12-myristate-13-acetate (PMA, an activator of protein kinase C for activating monocytes) were purchased from Selleck. RNAiso plus reagent was purchased from TAKARA.

### Virus infection

In this study ZIKV SZ01 strain (GenBank: KU866423), a gift from Dr Shibo Jiang of Fudan University, was used. THP-1 cells were activated with 100 nM PMA for 24 hrs, followed for another 24 hrs in resting culture without PMA [25]. THP-1 and RAW264.7 cells were inoculated with ZIKV SZ01 at an MOI of 1 for infection at 37°C for 12 to 48 hrs. Cell medium was collected and cell lysates or total RNA were prepared for further analyses.

### MTT assay for cell viability

PMA-activated THP-1 cells and RAW264.7 cells were inoculated with ZIKV (MOI 0.01, 0.1, or 1) for 12, 24, 36 and 48 hrs prior to addition of 3-(4,5)-dimethylthiahiazo (-z-y1)-3,5-di phenytetrazoliumromide (MTT) for another 4 hrs. Cell viability was determined as a ratio of absorbance at OD_570_ of ZIKV-infected cells to uninfected cells. The assay was carried out at least three times for each group. An unpaired Student’s t-test was used to evaluate the data. The data shown are the mean ± SD of three independent experiments (*P<0.05, ***P< 0.001).

### Quantitative realtime PCR

Total RNA was extracted with RNAiso plus reagent for quantitative real-time PCR (qPCR) following the manufacture’s manual. qPCR was performed with 1μl of cDNA in a total volume of 20μl with SYBR Green qPCR Master Mix (Vazyme) according to the manufacturer’s instructions. qPCR primers were designed by Primer Premier 5.0 and shown in S1 Table. Relative gene expression levels were normalized by β-actin housekeeping gene. Relevant fold change of each gene was calculated by following the formula: 2^ΔCt of gene-ΔCt of β-actin^ (ZIKV infected cells)/ 2^ΔCt of gene-ΔCt of β-actin^ (ZIKV—uninfected cells). One Step PrimeScript^™^ RT-PCR Kit Takara Bio, Japan) was used to quantify viral RNA copy numbers (TaqMan) in ZIKV-infected cell cultures, and the standard curves were generated using a plasmid encoding ZIKV E gene as a template with specific primers and probe (S1 Table).

#### Confocal immunofluorescence microscopy

1x10^5^ THP-1 cells, seeded on sterile coverslips in 24-well plates after stimulation with 100 nM PMA, were infected with ZIKV (MOI 0.1). The cells were fixed at 12 and 24 hrs post infection (p.i.) with 4% paraformaldehyde (Sigma-Aldrich, MO) for 15 min and permeabilized with 0.1% Triton X-100 for 10 min, followed by blocking with 5% BSA for 1 hr. The cells were incubated with an anti-ZIKV E mouse polyclonal antibody, 4G2 (1:200) and an anti-cleaved caspase 3 antibody (1:500) for 1 hr at room temperature. After four washes with PBS, the cells were incubated with AlexaFluor 488 labeled donkey anti-mouse IgG and AlexaFluor 594 labeled donkey anti-rabbit IgG (1:1000 each) for 1 hr at room temperature. After three washes with PBS, Antifade Mounting Medium with DAPI was applied to seal the coverslips, before the cells were subject to confocal laser scanning microscopy under an Olympus microscope (FLUOVIEW 3000).

As for TUNEL assay, the cells, infected or uninfected with ZIKV, were subject to TUNEL staining at various time points p.i. A TUNEL BrightGreen Apoptosis Detection Kit (Vazyme, China) was used for the assay following the protocol provided by the manufacturer.

#### Caspase enzymatic activity assay

Cell lysates were prepared from the infected or uninfected THP-1 cells, stimulated with PMA, and measured for specific caspase enzymatic activities. Caspase-8 and Caspase-9 Activity Assay Kits [26,27] were used following the protocol provided by the vendor. Cell lysates prepared from THP-1 cells, stimulated with TNF-α (20ng/mL) and Ginsenoside Rh2 (10 μg/mL) for caspase-8 and caspase-9, respectively, for 24 or 36 hrs, were also assayed as positive controls for caspase-8 or caspase-9 activity [28].

### Lentivirus packaging for shRNA

Lentivirus vectors were obtained from Shanghai Jiao Tong University. The negative control was a pLKO.1 vector containing sequences encoding shRNA. To ensure knockdown efficiency, we selected three shRNA sequences for each targeted gene. The sense sequences for pro-caspase-1 shRNA are: CTCTCATTATCTGCAATGA, AGCGTAGATGTGAAAAAAA, and CCAGATATACTACAACTCA. The sense sequences for NLRP3 shRNA are: TCGAGAATCTCTATTTGTA, ACGCTAATGATCGACTTCA, and AGGAGAGACCTTTATGAGA. PMA-activated THP-1 cells were infected with the recombinant lentiviruses expressing shRNAs targeting pro-caspase-1 or NLRP3. Forty-eight hours later, the culture medium was discarded and the cells were inoculated with ZIKV at an MOI of 1. The culture medium and cell lysates were harvested at indicated time points for enzyme-linked immunosorbent assay (ELISA) and western blot analyses.

### ELISA and western blot analysis

Secretion of cytokine IL-1β and IL-18 in culture medium was measured using commercial ELISA kits. Each group of testing was replicated for three times and resultant data were analyzed using the Student’s t-test. Cell lysates from the THP-1 (wild type, knockdown cells for NLRP3 and pro-caspase-1) and RAW264.7 (wild type and knockout cells for NLRP3) cells were prepared by RIPA lysis buffer with 1% PMSF. Protein concentrations were determined by a Bradford assay (BCA). Cell lysates (40 μg) were electrophoresed in 10–15% SDS-PAGE and the proteins transferred to a PVDF membrane for subsequent western blot analyses. Protein signals on the membrane were visualized by a GelCap ECL analyzer (Canon).

### Titration of infectious virus particles

Infectious particle titers in ZIKV-infected macrophages were determined by finding the Median Tissue Culture Infectious Dose (TCID50). Culture media from infected THP-1 or RAW264.7 cells were harvested at various time points p.i. for titration of titers on Vero E6 cells. 10-fold serially diluted culture medium was added to Vero E6 culture on 96-well plates and cell cytopathic effect was observed 4 or 5 days after the inoculation. The TCID50 of the infected cultures were calculated using the Reed-Muench method.

### Statistical analysis

The Student’s t-test was used to evaluate the data. The data shown are the mean ±SD of three independent experiments. The differences with a value of p<0.05 were considered statistically significant.

## Results

### Infection of macrophages by ZIKV triggered cytopathic effects

**Fig 1:**
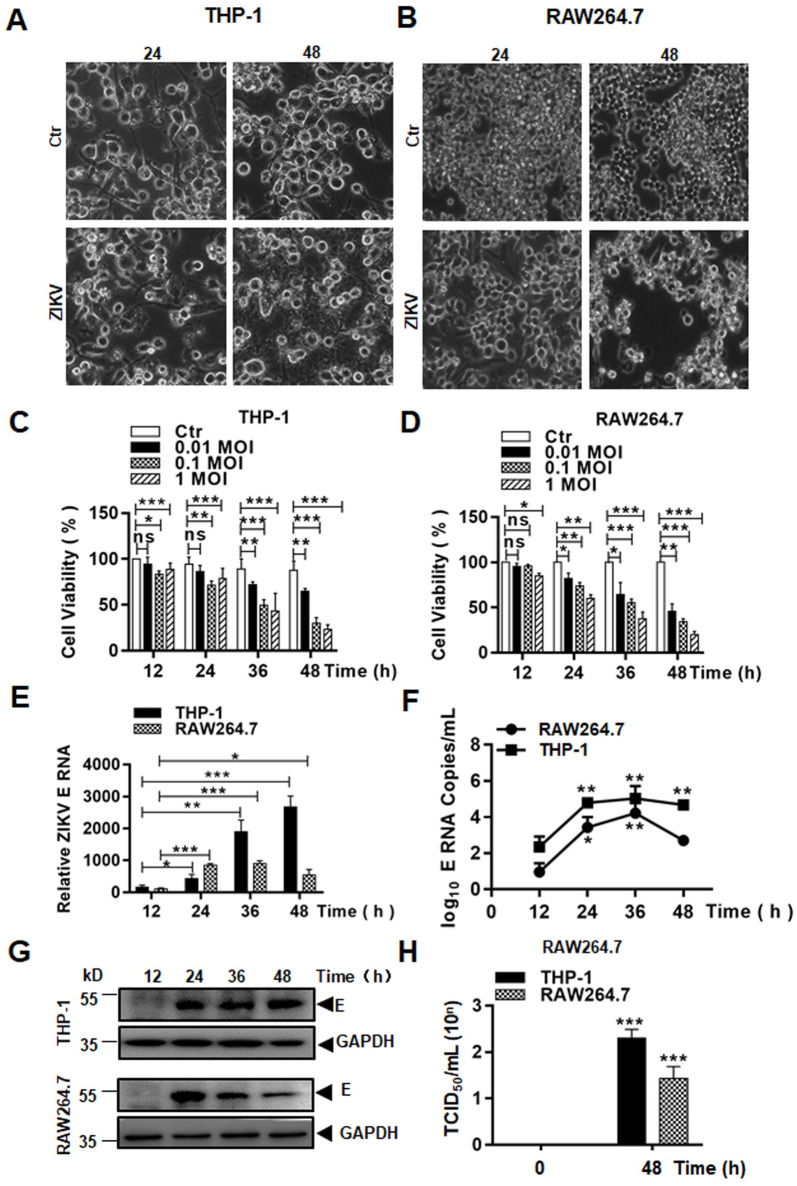
Viral replication and cell death caused in ZIKV-infected macrophages. Human THP-1 (**A**) and mouse RAW264.7 (**B**) cells were infected with ZIKV at 1 MOI and shown cytopathic effect (CPE) at 24 and 48 hrs p.i. (Magnification x40). Cell viability was assessed at various time points p.i. in THP-1 (**C**) and RAW264.7 (**D**) cells infected with ZIKV at various MOIs by an MTT assay. Total RNA was prepared for realtime RT-PCR to measure copy numbers of ZIKV E RNA in infected THP-1 and RAW264.7 cells. A SYBR Green approach for fold change (**E**) and a TaqMan approach (**F**) for viral RNA copy numbers were performed. (**G**) ZIKV E protein expression was detected in cell lysates prepared from THP-1 and RAW264.7 cells, infected with ZIKV at an MOI of 0.1, by western blot analyses. (**H**) Infectious viral titers (TCID50) were titrated in Vero E6 cells. The data were presented as mean ±SD and analyzed by Student’s t-test. *, P<0.05; **, P<0.01; ***, P< 0.001.

As an arbovirus, ZIKV enters bloodstream after the mosquito bite and may infect and replicate in some blood cell types. We chose to study macrophages, which are known to be susceptible to ZIKV, to examine the fate of infected cells and understand their role in the course of the infection. Two cell lines, THP-1 (human) and RAW264.7 (mouse), were selected for use in this study. Monocytic THP-1 cells were pre-treated with PMA for 24 hrs before the cells were fully activated and differentiated based on the upregulated transcription of CD14 and CD71 markers (S1A & S1B Fig). PMA-differentiated THP-1 and RAW264.7 cells were infected with ZIKV virus (MOI 0.1). Morphological changes were observed under a microscope, which showed that the cells became swollen and detached, and cell membranes ruptured (Fig 1A and 1B and S2 Fig). Increased secretion of lactate dehydrogenase (LDH) also confirmed the membrane rupture in ZIKV-infected macrophages (S1C Fig). Eventually the cell bodies disseminated into debris after 24 or 48 hrs p.i. The cell death caused by ZIKV infection was MOI dependent. When the cells were infected with the virus at MOIs of 0.01, 0.1, or 1, cell death occurred at various time points p.i. and more death was observed in the culture infected with higher viral doses (Fig 1C and 1D). To confirm that a productive infection occurred, we measured viral RNA replication which showed that viral E gene copy numbers increased over time p.i. by SYBR Green (Fig 1E) and TaqMan (Fig 1F) realtime RT-PCR in both THP-1 and RAW264.7 cells. Viral E protein was detected starting at 24 hrs p.i. in the infected cells (Fig 1G). Infectious virus particles were detected and titers determined in the ZIKV-infected macrophages with a TCID50 assay (Fig 1H).

### Programed cell death induced in macrophages with ZIKV infection

**Fig 2:**
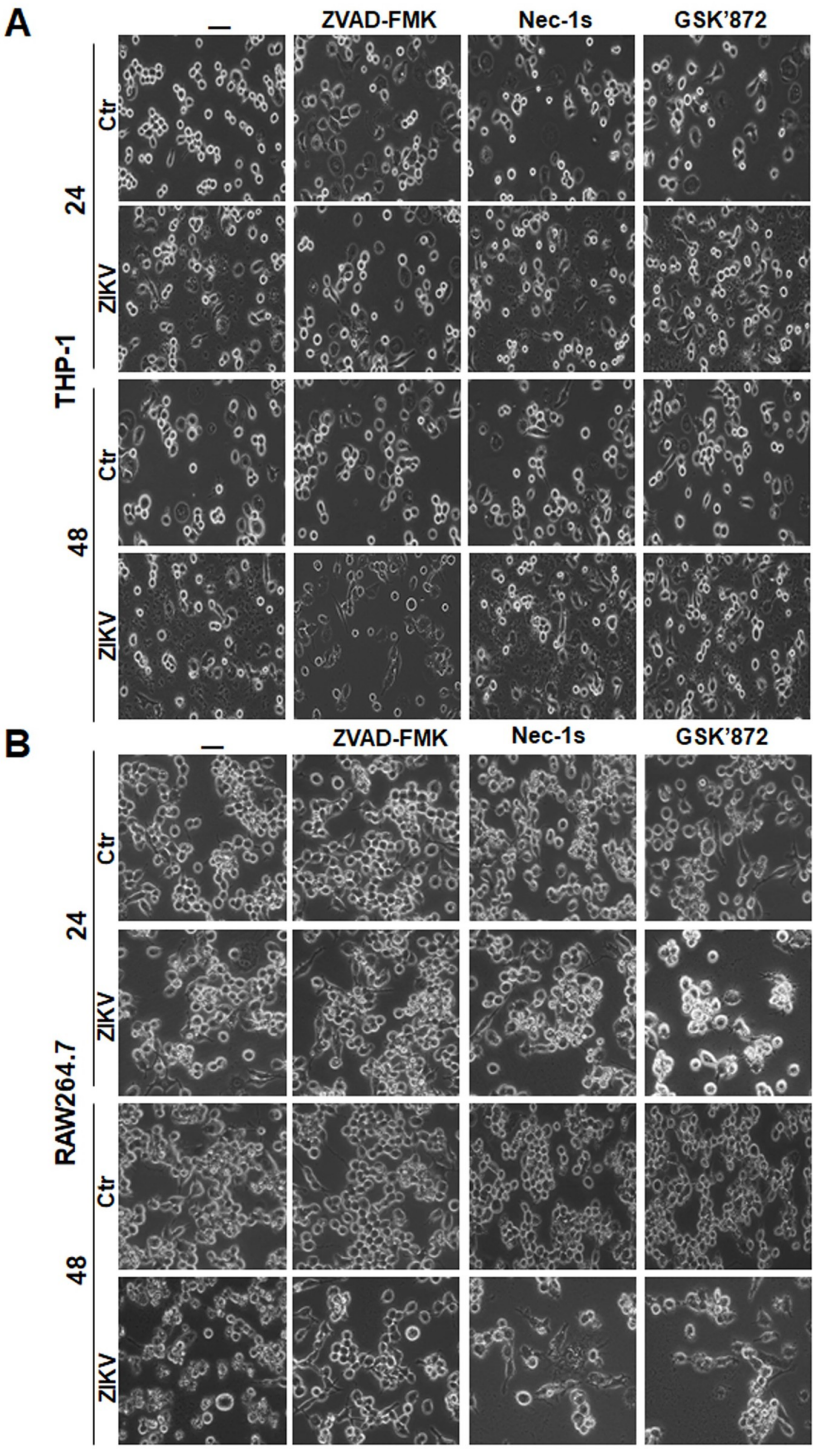
Morphological changes of cell death induced in ZIKV-infected monocytes pre-treated by programmed death inhibitors. THP-1 (**A**) and RAW264.7 (**B**) cells were pre-treated with pan-caspases inhibitor, ZVAD-FMK, or RIPK inhibitors, Nec-1s and GSK’872, followed by infection with ZIKV at 0.1 MOI. The cells were observed at 24 and 48 hrs p.i. under a light microscope (Magnification x200).

We attempted to elucidate the mechanism by which monocytes died in response to ZIKV infection. THP-1 and RAW264.7 cells were pre-treated with ZVAD-FMK (40 μM), Nec-1s (50 μM), and GSK’872 (20 nM), the inhibitors for pan-caspases, RIPK1, and RIPK3, respectively, followed by infection with ZIKV. First, we observed the morphological changes of infected cells with or without treatment of inhibitors. In the cells not treated with the inhibitors, infected cells underwent cell death. It appeared that ZVAD-FMK could relieve the cell death in both THP-1 and RAW264.7 cells, although neither Nec-1s nor GSK’872 had any apparent impact on cell death at 24 and 48 hrs p.i. (Fig 2A and 2B), indicating that caspases could be involved in the mechanism of cell death.

**Fig 3:**
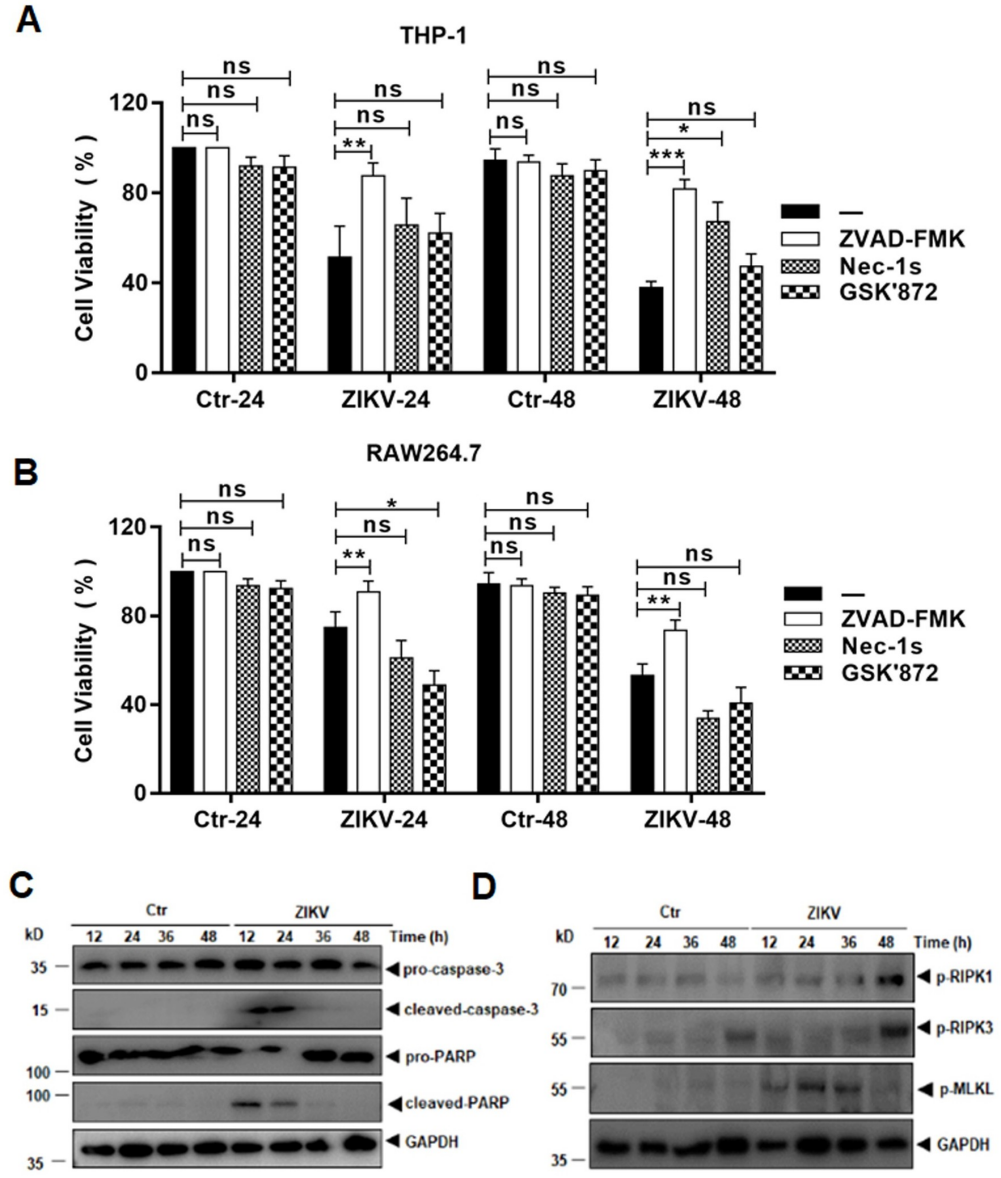
Blockage of cell death induced in ZIKV-infected cells by the inhibitors of programmed cell death. THP-1 (**A**) and RAW264.7 (**B**) cells were pre-treated with pan-caspases inhibitor, ZVAD-FMK, or RIPK inhibitors, Nec-1s and GSK’872, followed by infection with ZIKV at 1 MOI. The cell viability was assessed at 24 and 48 hrs p.i. with an MTT assay. The data were shown as mean ±SD and analysed by unpaired Students t-test. *, P<0.05; **, P<0.01; ***, P<0.001. ns, no significance. (**C**) Cleavage of pro-caspase-3 and PARP in ZIKV-infected THP-1 cells as detected in a western blot analysis. (**D**) Phosphorylation of RIPK and MLKL in ZIKV-infected THP-1 cells.

Cell death was quantified by measuring cell viability with an MTT assay. The treatment with ZVAD-FMK significantly prevented monocyte cell death at 24 and 48 hrs p.i. in both THP-1 (Fig 3A) and RAW264.7 (Fig 3B) cells. On the other hand, treatment with Nec-1s did not prevent cell death until 48 hrs p.i. in THP-1 cells and had no effect on cell death in RAW264.7 cells. Treatment with GSK’872 did not reverse cell death in either THP-1 or RAW264.7 cells. Taken together, these data suggest that caspases, but not RIPKs, may play a role in the cell death triggered by ZIKV infection.

We analysed the cell lysates prepared at various time points p.i. from the infected cells with western blot analyses. As shown in Fig 3C, pro-caspase 3 was cleaved, activated and detected, together with the cleaved substrate PARP, at the early stage of infection, confirming that apoptosis was activated in ZIKV-infected macrophages, although other caspase-dependent cell death was not excluded. We could also detect increased phosphorylation of RIPK1 and RIPK3, as well as phosphorylated MLKL. However, the activation of RIPKs may not have efficiently triggered a programed necrosis. These data suggest that caspases-dependent programed cell death, including apoptosis, could be the main cause involved in ZIKV-infected macrophages.

### Intrinsic apoptosis was induced in ZIKV-infected human macrophages

**Fig 4:**
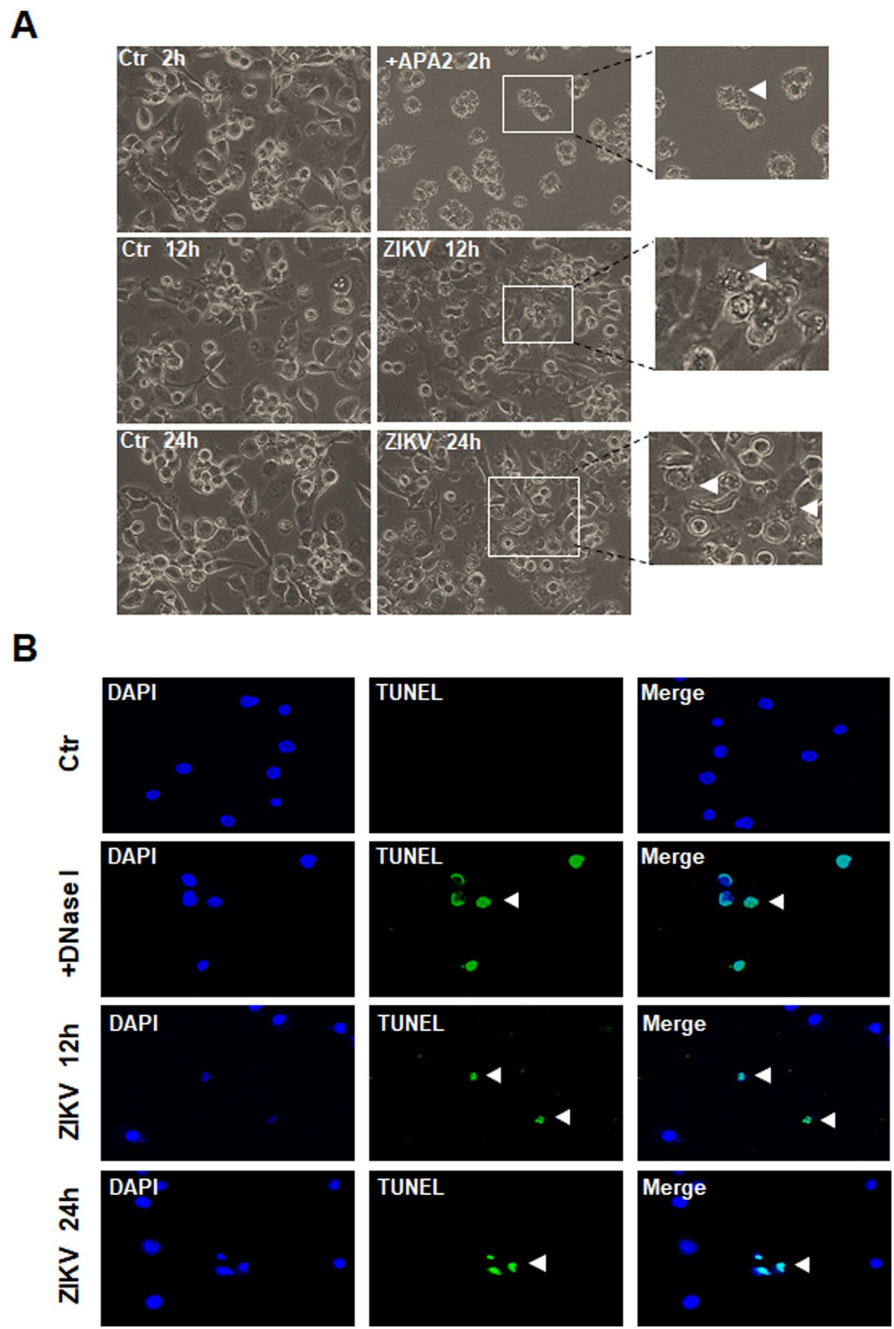
ZIKV infection induced apoptosis in macrophages. PMA-differentiated THP-1 cells were infected with ZIKV and observed under a light microscope at 12 and 24 hrs p.i. for the cells showing apoptotic blebing (arrow) (**A**). The cells were treated with 10 μM of apoptosis activator 2, a caspase3 activator, for 2 hrs as a positive control. A TUNEL assay was applied to ZIKV-infected THP-1 cells at 12 and 24 hrs p.i. and the TUNEL positive cells were shown (arrow). The cells were treated with 20 U/mL of DNase I for 10 min as a positive control (**B**).

**Fig 5:**
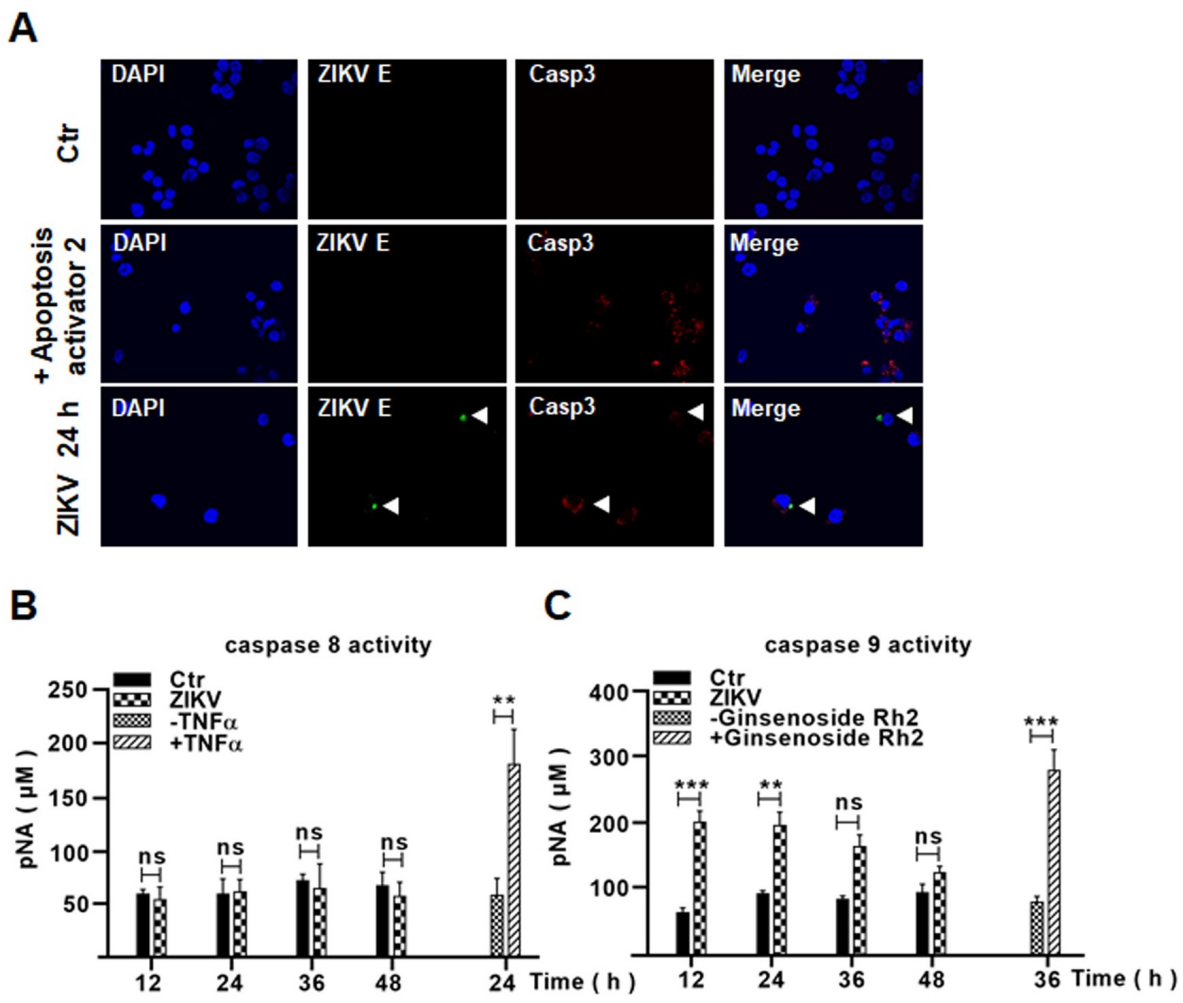
ZIKV infection triggered the intrinsic apoptosis in ZIKV-infected macrophages. (**A**) PMA-differentiated THP-1 macrophages, infected with 0.1 MOI of ZIKV for 24 hrs before staining with antibodies for ZIKV E and cleaved caspase-3 and subjected to confocal immunofluorescence. The infected cells positive with ZIKV E and cleaved caspase-3 were shown (arrow). The cells were treated with 10 μM of apoptosis activator 2 for 2 hrs as a positive control for apoptosis. (**B** & **C**) Activation of caspase-9 in ZIKV-infected macrophages. Cell lysates were prepared from ZIKV-infected THP-1 cells for assay of caspase-8 (**B**) and caspase-9 (**C**) enzymatic activities. The cells were treated with either 20 ng/mL of TNF-α for 24 hrs or 10 μg/mL of Ginsenoside Rh2 for 36 hrs as positive controls for activities of caspase-8 or caspase-9. The experiments were performed in triplicates and the data were shown as mean+SD, analyzed by unpaired Students t-test. **, P<0.01; ***, P<0.001. ns, no significance.

We further studied apoptotic processes induced in ZIKV-infected THP-1 cells and apoptotic blebbing was observed in some infected cells (Fig 4A). In cultures which were infected with ZIKV as E protein was expressed, TUNEL positive cells were detected 12 and 24 hrs p.i. (Fig 4B). Cleaved caspase-3 was demonstrated in ZIKV-infected cells, too, as shown by confocal immunofluorescence microscopy (Fig 5A). Cell lysates were prepared from ZIKV-infected macrophages for a caspase enzymatic activity assay, which indicated that only caspase-9 activity was detected, while no caspase-8 activity was detectable (Fig 5B and 5C). These data collectively demonstrated that a caspase-9 dependent intrinsic apoptosis was activated in ZIKV-infected macrophages.

### Pyroptosis was induced in both human and murine macrophages during ZIKV infection

**Fig 6:**
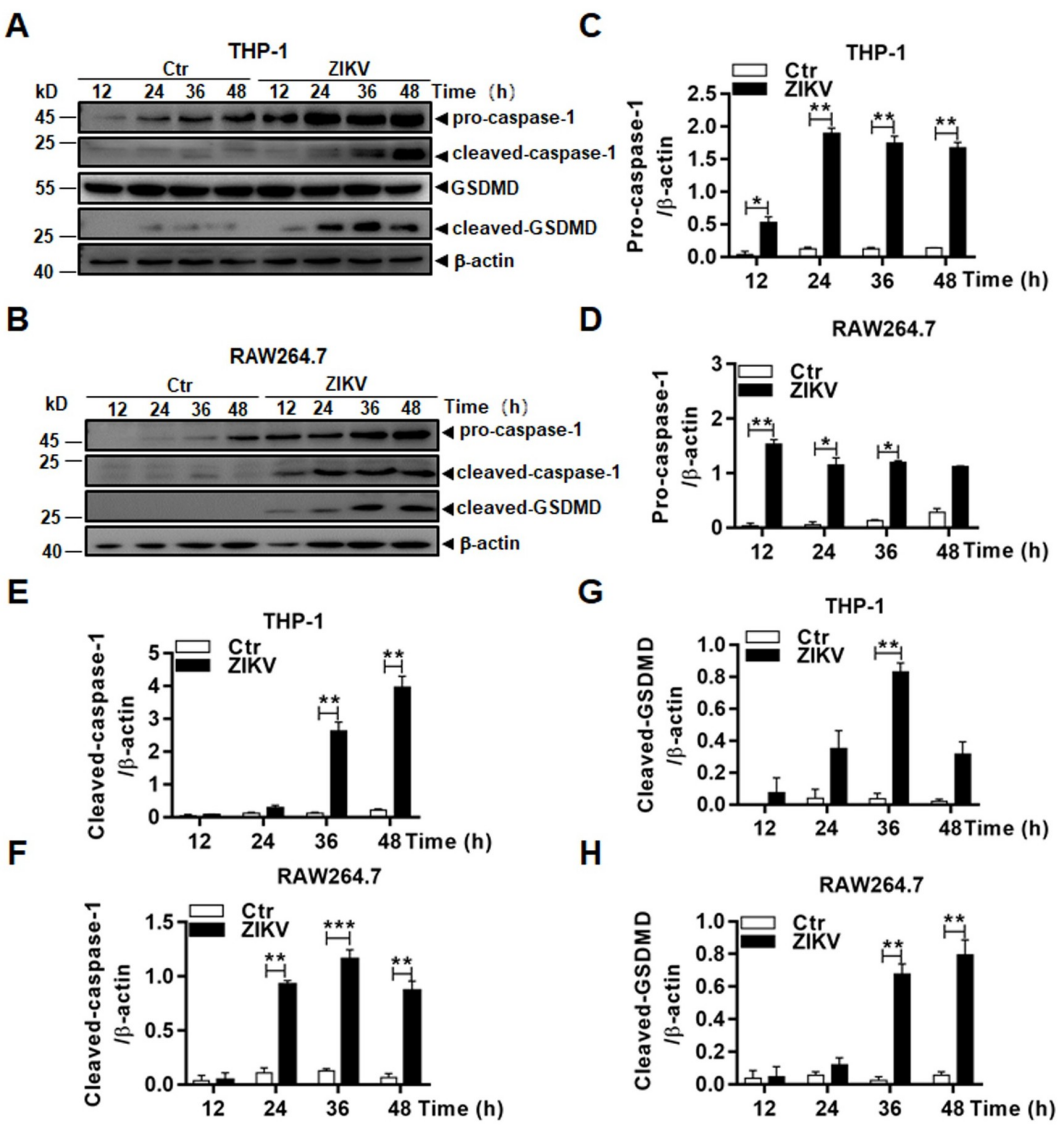
Inflammasome activation led to pyroptosis in ZIKV-infected macrophages. Cell lysates, prepared at various time points p.i. from THP-1 (**A**) and RAW264.7 (**B**) cells infected with 0.1 MOI of ZIKV, were analysed by western blot analyses with antibodies for pro- or cleaved caspase-1 and GSDMD. Quantitative analyses of the grayscale values in the blots of the pro- and cleaved caspase-1 and GSDMD in infected and control THP-1 (**C**, **D**, & **F**) and RAW264.7 (**E**, **G**, & **H**) cells were shown. The experiments were performed in triplicates and the data were shown as mean+SD, analyzed by unpaired Students t-test. *, P<0.05; **, P<0.01.

Considering that caspases are involved in not only apoptosis but also pyroptosis, an inflammatory type of cell death, we decided to examine what types of caspases were required for the cell death in ZIKV-infected macrophages. Pro-caspase-1 was activated as shown in the early studies indicating that inflammasomes were activated in macrophages [15,16]. We confirmed that in both THP-1 and RAW264.7 cells (Fig 6A and 6B) pro-caspase-1 was processed and that cleaved caspase-1 was detected at various time points p.i. by western blot analyses. In fact, expression of pro-caspase-1 was upregulated in both cell lines after ZIKV infection. In addition, GSDMD, a substrate of caspase-1, was cleaved to become cleaved GSDMD, an executor of pyroptosis, indicating that ZIKV infection activated inflammasomes, and induced pyrotosis, probably via a caspase-1-dependent canonical pathway. Quantitative analyses showed significant upregulation of pro-caspase-1 (Fig 6C and 6D) and an increase of cleaved caspase-1 (Fig 6E and 6F) and GSDMD (Fig 6G and 6H) in infected THP-1 and RAW264.7 cells over levels in uninfected cells.

ZIKV-infected macrophages were further analyzed, which showed that the transcription of HLA-DRA, but not CD163, was upregulated indicating that ZIKV infection polarized THP-1-differentiated macrophages (M0) to proinflammatory M1 phenotype macrophages (S1B Fig). Increased secretion of lactate dehydrogenase (LDH) confirmed the assumption that the cell death of ZIKV-infected macrophages was both inflammatory and necrotic (S1C Fig).

### Upregulation of the components for inflammasome formation and its activation in ZIKV-infected macrophages

**Fig 7:**
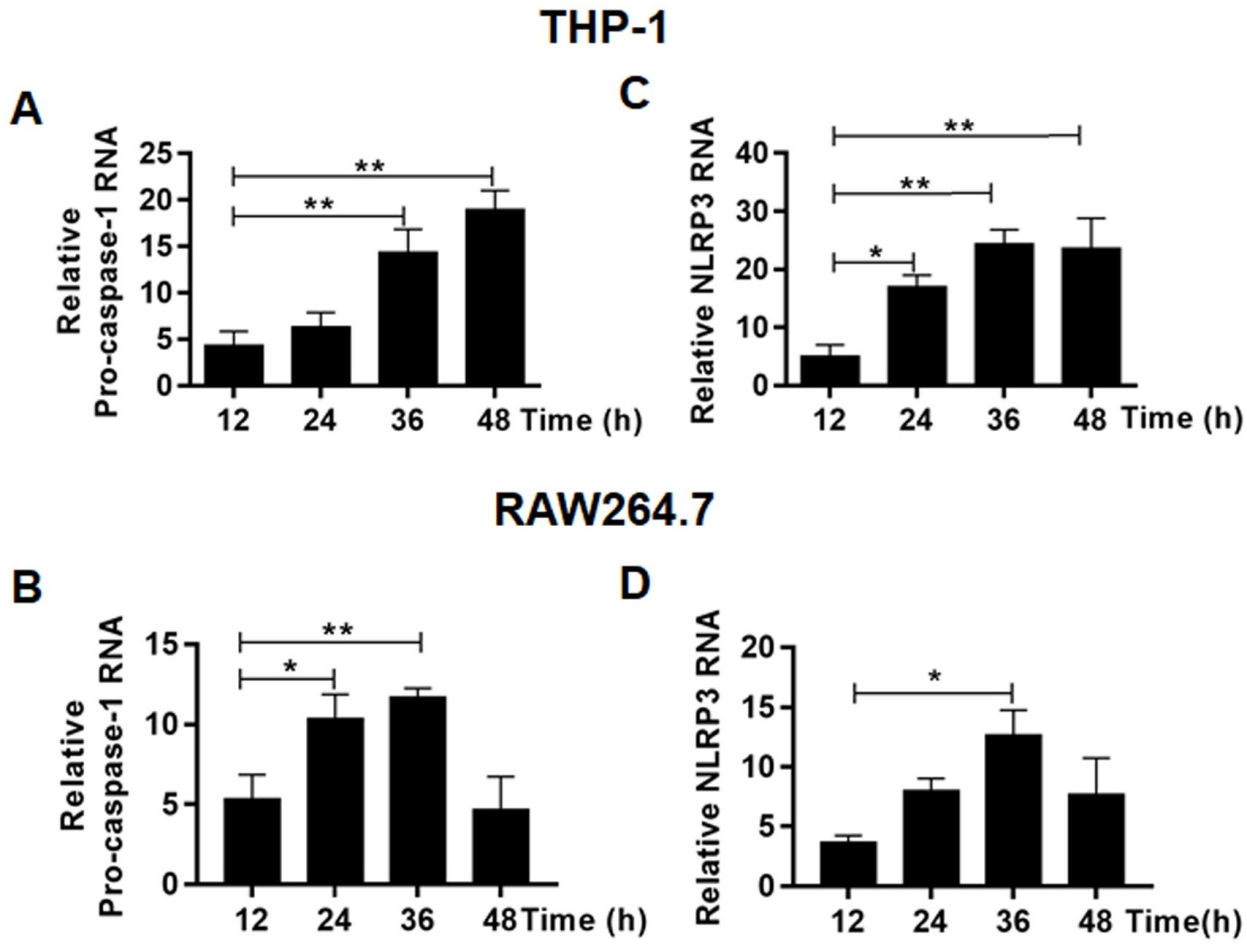
Upregulation of the inflammasome components in ZIKV-infected macrophages. Total RNA was prepared at various time points p.i. from THP-1 and RAW264.7 cells infected with 0.1 MOI of ZIKV for realtime RT-PCR to measure mRNA transcript numbers of pro-caspase-1 (**A** & **B**) and NLRP3 (**C** & **D**). The experiments were performed in triplicates and the data were shown as mean+SD, analysed by unpaired Students t-test. *, P<0.05; **, P<0.01. ns, no significance.

**Fig 8:**
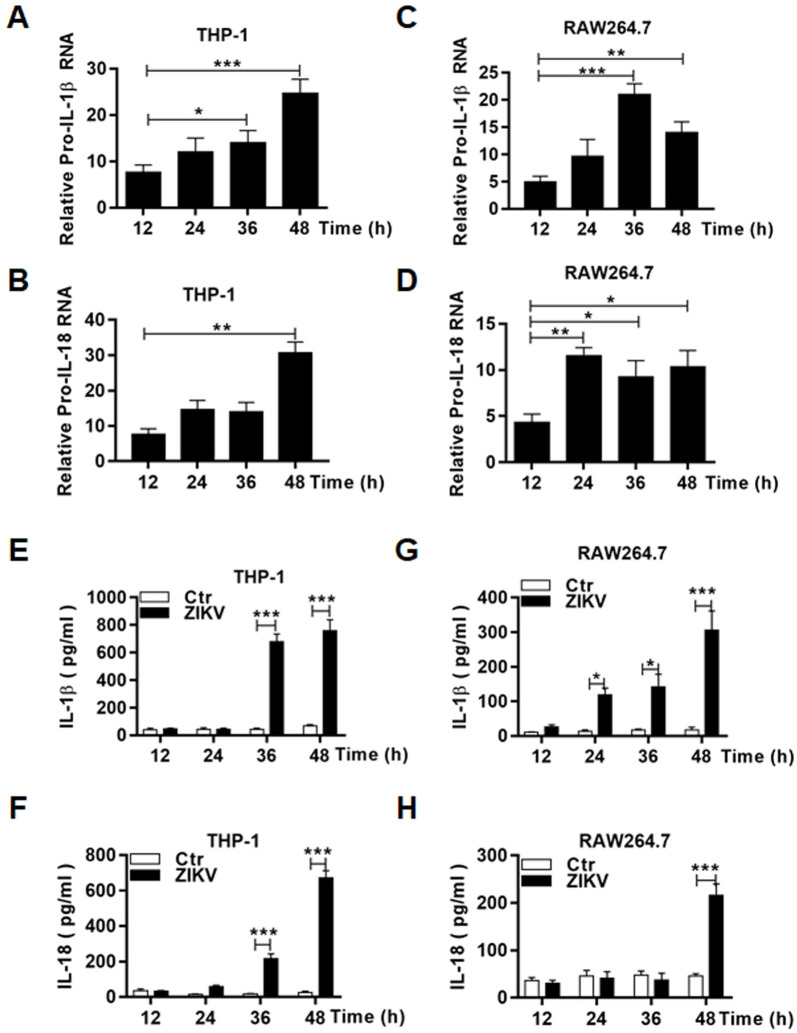
Transcriptional upregulation and secretion of IL-1β and IL-18 in ZIKV-infected human and murine macrophages. Total RNA was prepared at various time points p.i. from THP-1 (**A-B**) and RAW264.7 (**C-D**) cells infected with 0.1 MOI of ZIKV for realtime RT-PCR to measure mRNA transcript numbers of pro-IL-1β (**A** & **C**) and pro-IL-18 (**B** & **D**) genes. Culture medium was sampled at various time points p.i. from ZIKV-infected THP-1 (**E-F**) and RAW264.7 (**G-H**) cells for measurement of secreted IL-1β (**E** & **G**) and IL-18 (**F** & **H**) by ELISA. The experiments were performed in triplicates and the data were shown as mean+SD and analysed by unpaired Students t-test. *, P<0.05; **, P<0.01; ***, P<0.001. ns, no significance.

We examined the transcription and post-transcriptional processing of inflammasome components in infected THP-1 cells. RNA transcripts of pro-caspase-1 (Fig 7A & 7B), NLRP3 (Fig 7C & 7D) genes increased significantly over the time p.i. in both human and murine macrophages infected with ZIKV. We also examined the transcripts of pro-IL-1β and pro-IL-18 and found their RNA copy numbers increased as well in infected THP1 (Fig 8A and 8B) and RAW264.7 cells (Fig 8C and 8D). We confirmed that IL-1β and IL-18 were released to the culture medium of the infected THP-1 (Fig 8E and 8F) and RAW264.7 (Fig 8G and 8H) cells by ELISA.

### Inflammasome activation led to cell death induced by ZIKV in macrophages

**Fig 9:**
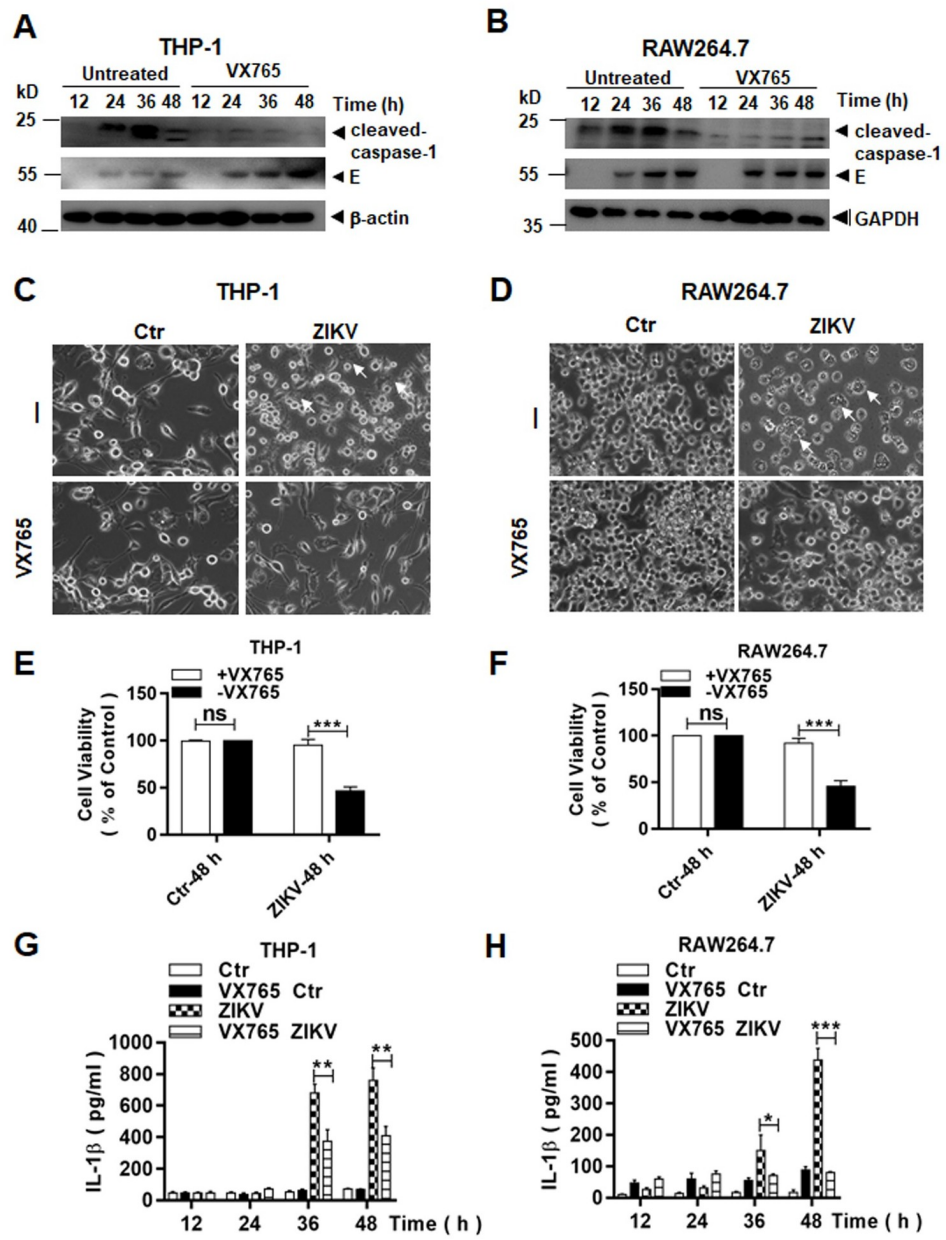
Pyroptosis was dependent on caspase-1 in ZIKV-infected human and murine macrophages. Cell lysates were prepared at various time points p.i. from ZIKV-infected THP-1 (**A**) and RAW264.7 (**B**) cells which were untreated or pre-treated with VX765, a caspase-1 inhibitor, at 20μM. The lysates were analysed by western blot analyses with an anti-cleaved caspase-1 antibody. Morphological changes were observed on ZIKV-infected THP-1 (**C**) and RAW264.7. Arrows, swollen or ruptured cells. (**D**) cells, which were untreated or pre-treated with VX765, under a light microscope (Magnification x200). (**E** & **F**) Cell viability of THP-1 and RAW264.7, pre-treated with or without VX765 and followed by ZIKV infection (0.1 MOI), was measured by a CCK8 assay. (**G** & **H**) Culture medium was sampled at various time points p.i. from ZIKV-infected THP-1 and RAW264.7 cells, which were untreated or pre-treated with VX765, for measurement of secreted IL-1β by ELISA. The experiments were performed in triplicates and the data were shown as mean+SD and analyzed by unpaired Students t-test. *, P<0.05; **, P<0.01; ***, P<0.001. ns, no significance.

Pyroptosis can be triggered by processing of pro-caspase 1 via a canonical inflammasome activation, or processing of pro-caspase 4, 5, or 11 via a non-canonical approach. To confirm the mechanism for pyroptosis that occurred in macrophages infected with ZIKV, we chose to use Belnacasan, or VX-765, a specific caspase-1 inhibitor to pre-treat the cells prior to viral infection. Cell lysates were prepared at various time points p.i. for western blot analyses. As shown in Fig 9, cleaved caspase-1 did not appear or the level of cleaved caspase-1 was greatly reduced in VX765-treated macrophages, in comparison to cells without treatment, indicating that VX765 effectively suppressed activation of pro-caspase-1 in infected THP-1 (Fig 9A) and RAW264.7 (Fig 9B) cells. We examined the morphology of infected THP-1 and RAW264.7 cells at 48 hrs p.i. with or without VX765 treatment. The number of swollen or ruptured cells decreased significantly in both THP-1 (Fig 9C) and RAW264.7 (Fig 9D) cells, which were pre-treated with VX756. We performed a quantitative analysis of cell viability, which clearly showed significantly more viable THP-1 (Fig 9E) and RAW264.7 (Fig 9F) cells in cultures pre-treated with VX765, indicating that pro-caspase-1 cleavage or activation of inflammasomes was critical to triggering pyroptosis in ZIKV-infected macrophages.

To ascertain the inflammasome activation to be inhibited, we detected the secretion of IL-1β in infected macrophages, pre-treated with VX765 or not. The increase of IL-1β in infected cells was significantly suppressed at 36 and 48 hrs p.i. in VX765 pre-treated cells, compared to the cells without the treatment (Fig 9G and 9H).

**Fig 10:**
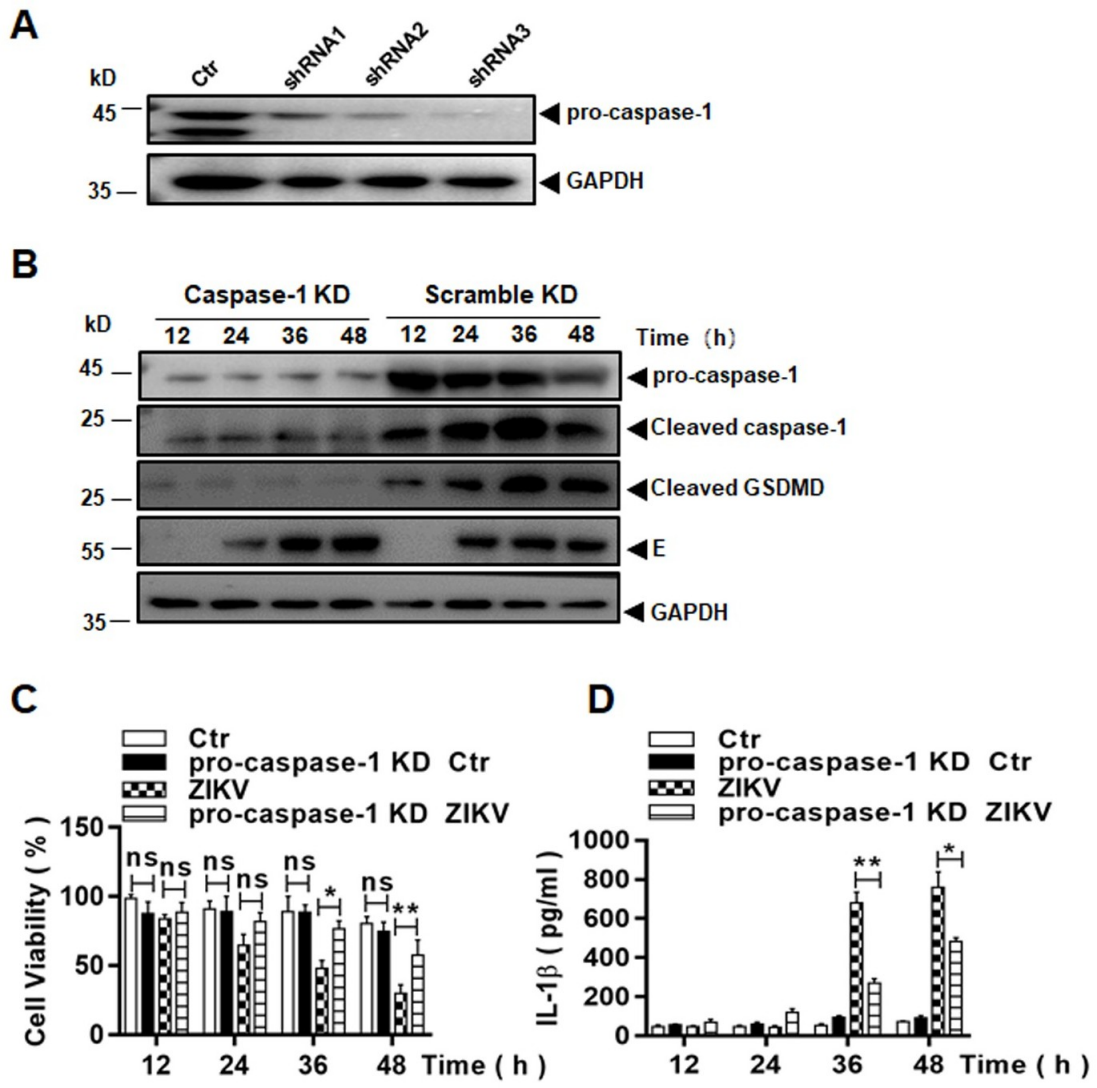
Pyroptosis was suppressed in ZIKV-infected macrophages with pro-caspase-1 knockdown. (**A**) Knockdown of pro-caspase-1 in THP-1 cells. Cell lysates were prepared from lentiviral vector-transduced THP-1 cell lines, expressing shRNA1, 2, or 3 targeting mRNA of pro-caspase-1, for western blot analyses with pro-caspase-1 antibody to examine the knockout (KD) efficacy. (**B**) Inhibition of pro-caspase-1 and GSDMD processing in pro-caspase-1 KD cells. Both pro-caspase-1 or scramble shRNA KD cells were infected with 0.1 MOI of ZIKV and cell lysates, prepared at various time points p.i., were subjected to western blot analyses with antibodies for pro-caspase-1, cleaved caspase-1 and GSDMD. (**C**) Cell viability was assessed in pro-caspase-1 or scramble shRNA KD THP-1 cells infected with or without ZIKV at various MOIs by an MTT assay. (**D**) Culture medium was sampled at various time points p.i. from pro-caspase-1 or scramble shRNA KD cells infected with or without ZIKV for measurement of secreted IL-1β by ELISA. The experiments were performed in triplicates and the data were shown as mean+SD and analysed by unpaired Students t-test. *P, <0.05; **, P<0.01. ns, no significance.

We further used a small hairpin RNA (shRNA) approach to confirm the role of inflammasome activation involved in cell death. Three shRNA molecules, targeting pro-caspase-1, were tested in THP-1 cells to knock down pro-caspase-1 transcription. The expression of pro-caspase-1 was suppressed in the cell lines, which were selected and expanded, as shown in Fig 10A. The caspase-1 knockdown (KD) cell lines were infected with ZIKV and cell lysates were prepared at various time points p.i. for western blot analysis. As shown in Fig 10B, little expression of pro-caspase-1, barely detectable cleaved caspase-1, and no detectable cleaved GSDMD were demonstrated, a pattern distinct from those in the control cells infected with ZIKV. Cell viability was measured by an MTT assay and significant cell death occurred in ZIKV-infected cells at 36 and 48 hrs p.i. However in pro-caspase-1 KD cells, the cell death was reversed significantly in the absence of pro-caspase-1 (Fig 10C), indicating that the pyroptosis, that occurred in ZIKV-infected macrophages, was caspase-1 dependent via a canonical pathway.

To ascertain the effect of pro-caspase-1 KD on inflammasome activation, we measured IL-1β expression and found it to be significantly suppressed at 36 and 48 hrs p.i., compared to the infected control cells without pro-caspase-1 KD, confirming that the pro-caspase-1 KD could suppress inflammasome activation in ZIKV-infected macrophages (Fig 10D).

### The NLRP3 inflammasome was essential to ZIKV-induced pyroptosis

**Fig 11:**
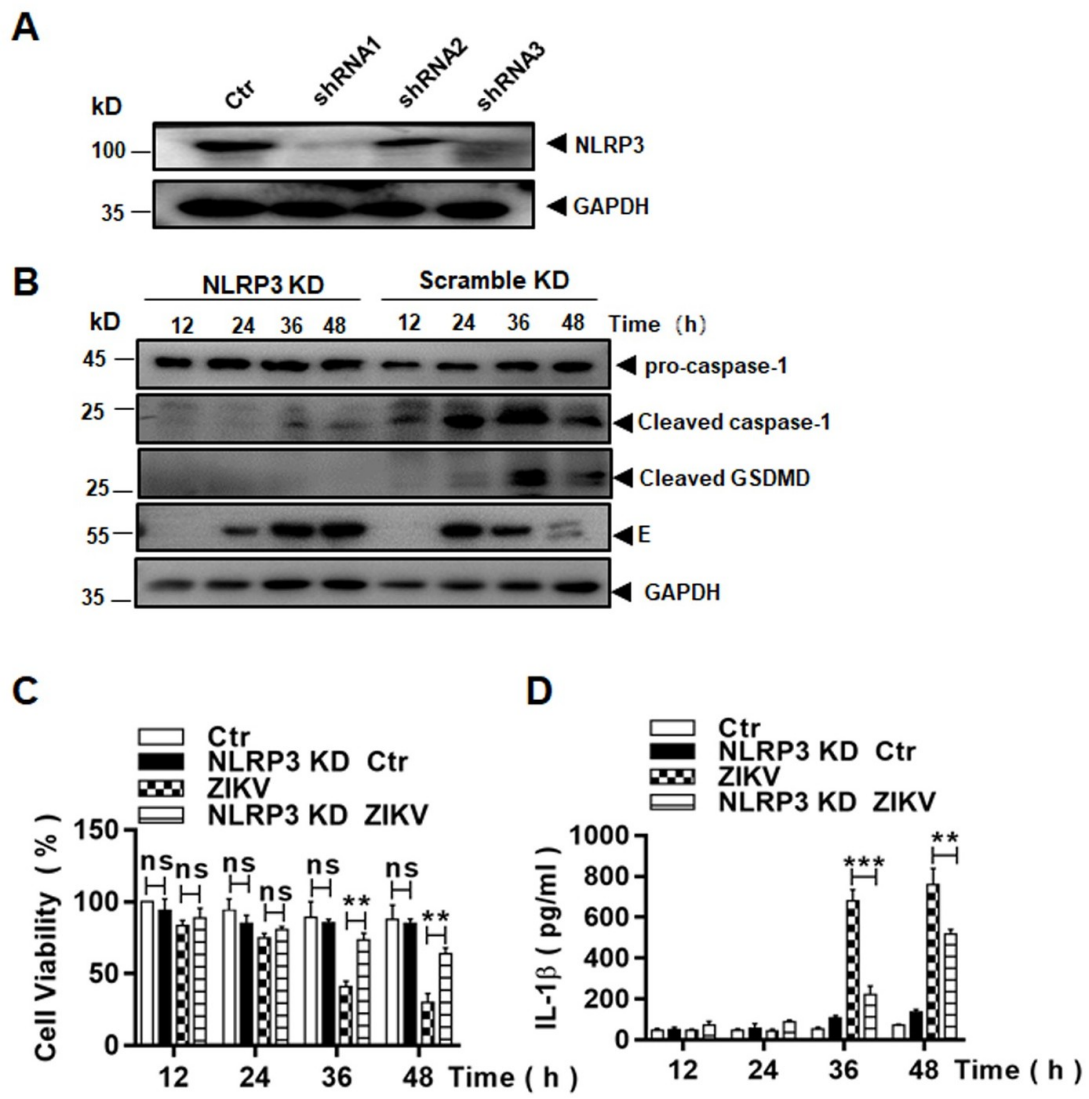
Pyroptosis triggered in ZIKV-infected monocytes were dependent on the NLRP3 inflammasome activation. (**A**) Knockdown of NLRP3 in THP-1 cells. Cell lysates were prepared from lentiviral vector-transduced THP-1 cell lines, expressing shRNA1, 2, or 3 targeting mRNA of NLRP3, for western blot analyses with an NLRP3 antibody to examine the knockdown (KD) efficacy. (**B**) Inhibition of pro-caspase-1 and GSDMD processing in NLRP3 KD cells. Both NLRP3 or scramble shRNA KD cells were infected with ZIKV and cell lysates, prepared at various time points p.i., were subjected to western blot analyses with antibodies for pro-caspase-1, cleaved caspase-1 and GSDMD. (**C**) Cell viability was assessed in NLRP3 or scramble shRNA KD THP-1 infected with or without ZIKV at various MOIs by an MTT assay. (**D**) Culture medium was sampled at various time points p.i. from NLRP3 or scramble shRNA KD cells, infected with or without ZIKV, for measurement of secreted IL-1β by ELISA. The experiments were performed in triplicates and the data were shown as mean+SD and analysed by unpaired Students t-test. **, P<0.01; ***, P<0.001. ns, no significance.

As described earlier, NLRP3 was transcriptionally upregulated in human macrophages infected with ZIKV. To confirm the role of the NLRP3 inflammasome activation in pyroptosis, we carried out an shRNA knockdown of NLRP3 in THP-1 cells. Three shRNA molecules with different sequences, targeting NLRP3, were tested in THP-1 cells to knock down NLRP3 transcription. The cell lines were selected and examined for their knockdown efficacy. The resultant knockdown of NLRP3 efficacy is shown in Fig 11A. The NLRP3 knockdown (KD) cells were infected with ZIKV and cell lysates were prepared for western blot analysis. In infected control cells, pro-caspase-1 was processed and cleaved, GSDMD was produced, but in infected KD cells, pro-caspase-1 was barely processed and cleaved and GSDMD was not produced (Fig 11B). This indicates that pyroptosis, triggered in infected monocytes, was dependent on NLRP3 inflammasome activation, a canonical approach, leading to processing of pro-caspase 1.

Cell viability was measured with an MTT assay in NLRP3 KD cells after infection. As shown in Fig 11C, significant cell death occurred at 36 and 48 hrs p.i. in infected control cells but the cell death was effectively suppressed in the NLRP3 KD cells infected with ZIKV.

To confirm that NLRP3 inflammasome activation was impaired in the NLRP3 KD cells, we further measured the secretion of IL-1β in infected control and NLRP3 KD cells by ELISA, which showed that the increase of IL-1β secretion in the culture medium was significantly reduced in the NLRP3 KD cells (Fig 11D). In sum, these data demonstrate that the activation of the NLRP3 inflammasome led to pyroptosis, which was dependent on activated caspase-1 in ZIKV-infected macrophages.

## Discussion

ZIKV causes asymptomatic or mild infections, which are self-limited in most adults, indicating that host immunity can contain the infection effectively in most adults. However, ZIKV can disseminate through bloodstream to the placenta in some pregnant women and penetrate the blood-placenta barrier (BPB) into fetuses, in which the virus invades the neural tissues due to its neurotropism. What role monocytes or macrophages play in facilitating viral penetration of the BPB barrier and eventually in infection of the fetal brain remains unknown. In this study, we confirmed that both human and murine macrophages were susceptible to ZIKV, and a productive replication led to cell death. The cell death was observed at 12 hrs p.i., and continued to eventually deteriorate the whole cell culture, suggesting that ZIKV caused a lytic infection in macrophages in addition to causing the release of proinflammastory cytokines and chemokines as shown in this and previous studies. We were able to identify that apoptotic process was triggered upon ZIKV infection, which also occurs in macrophages infected with many types of viruses [29–33]. In this study the similar outcome was observed in ZIKV-infected human and murine macrophages, indicating that it could be a common response with concomitant apoptotic and pyroptotic cell death to occur in macrophages infected with ZIKV.

Previous studies have shown that ZIKV infection activates the NLRP3 inflammasome, which leads to processing of pro-caspase-1 and secretion of IL-1β in infected monocytic cell lines, PBMC, or monocyte-derived macrophages [14–16,18]. Interestingly, none of these studies have pursued pyroptosis induction in infected monocytes or macrophages. In this report we showed the occurrence of both pyroptosis and apoptosis, in ZIKV-infected monocyte-derived macrophages. As reported previously, we showed that pro-caspase-1 was cleaved and the secretion of cytokines IL-1β and IL-18 increased, indicating that inflammasomes were activated in macrophages. Cleaved GSDMD, an executor of pyroptosis, was further detected, suggesting that a considerable amount of cell death in ZIKV-infected macrophages can be attributed to pyroptosis, which may be suppressed in cells pre-treated with an inhibitor for caspase-1 or shRNA to knockdown caspase-1. Finally, we showed that NLRP3 inflammasome activation was required for pyroptosis via a canonical approach, which was dependent on caspase-1 in ZIKV-infected human and murine macrophages [34].

Monocytes and macrophages are important in viral infections. As haemopoietic cells originating in the bone marrow, monocytes comprise about 10% of blood leukocytes. They are released into peripheral circulation and live for a few days in blood vessels of the body. Monocytes penetrate through the wall of vessels into tissues and organs through chemotaxis when microbial infection occurs, and differentiate into macrophages. Monocytes are susceptible to many viruses from various families. Human monocyte-derived macrophages can be infected by Coxsackieviruses CV-B4 [35], which, however, can poorly infect human monocytes, unless a non-neutralizing anti-CV-B4 IgG is present [36]. Replication of neurovirulent poliovirus strains in monocytes was associated to their pathogenesis in the central nervous system [37]. Monocytes and macrophages play critical roles in HIV transmission, viral spread early in the host, and being a reservoir of virus throughout infection [8].

Circulating monocytes are one of the early cellular targets of ZIKV infection in humans. Not only can monocytes be infected dominantly in PBMC by ZIKV in vitro, but the virus can also be found in monocytes collected during acute illness from Zika patients [12,38]. ZIKV viral RNA can be detected in the monocytes longer than in the serum of infected patients, indicating that monocytes could serve as a virus reservoir during the infection [38]. Interestingly, infection of monocytes by ZIKV leads to cell expansion to become more intermediate or a non-classical type by expressing CD16 [12,38]. Moreover, monocytes infected with ZIKV tend to secrete IL-10, an immunosuppressive cytokine, which could skew the host immune response during the early stage of pregnancy [12]. These reports collectively support that monocytes may play a complicated role in viral pathogenesis on ZIKV spread and neuropathogenesis. In this sense, programed cell death in ZIKV-infected monocytes, not reported previously to our knowledge, may be beneficiary to the host as a protective defence by eliminating the virus reservoir in the host.

Programed cell death can be triggered in monocytes infected with other flaviviruses. It has been observed that dengue virus (DENV) can induce apoptosis in infected monocytes, which is related to increased TNF-α induction [33]. In fact all four serotypes of DENV can induce apoptosis in human monocytes, dependent on activation of caspase 7, 8, and 9 [39]. On the other hand, DENV can also trigger pyroptosis, which is associated with an activation of caspase-1 and release of IL-1β [40]. In this report our data demonstrate that a concomitant apoptosis and pyroptosis were induced in ZIKV-infected human and murine macrophages. It was reported that NLRP3 inflammasome activation, triggered by ZIKV infection in monocytes, promotes the cleavage of cGAS, resulting in the inhibition of initiating type I IFN signaling, and enhances viral replication [14]. We believe that subsequent pyroptotic cell death, caused by NLRP3 inflammasome activation, or caspase-3 dependent apoptosis as shown by our data may help shorten or clear viral replicative niches and rid the host of viral carriers. Programed cell death may be also significant in preventing the virus from spreading to the placenta and infecting fetal chorionic villi during pregnancy.

Little is known about why an infected cell dies one way or another. In contrast to apoptosis, pyroptosis, and necroptosis are inflammatory, leading to massive damage of involved tissues. Different stimuli, occurring in the infected cells, may determine a specific death program. The endoplasmic reticulum (ER) stress may lead to apoptosis with the upregulation of the C/EBP homologous protein (CHOP) in ZIKV infected epithelial cells. But ZIKV could also impair the expression of CHOP and delay the ER-stress driven apoptosis [41]. Depending on its early or delayed occurrence, ZIKV-induced apoptosis could impose a duality of the effect in viral pathogenesis [42]. Pro-apoptotic factors in ZIKV-infected neuroprogenitor cells lead to induce early apoptosis, which may contribute to congenital defects in the brain of newborns. Other factors including upregulation of anti-apoptotic Bcl-2 or inhibition of the CHOP function can delay apoptosis in the reproductive or genital tract. Thus the delayed apoptosis may benefit ZIKV persistent infection for sexual transmission [42].

Cross-talk occurs between signaling pathways of programmed cell deaths, which may provide a mechanism for regulating cell fate. It was recently reported that some virus infection can cause a mode of complicated programed cell death, PANoptosis, which engages key molecules from pyroptosis, apoptosis, and/or necroptosis [43,44]. PANoptosis occurred in influenza A virus-infected macrophages characteristic of a multi-protein complex formation, containing ZBP1, RIPK3, RIPK1, caspase-8, ASC, and NLRP3 and defined as PANoptosome [43]. We have identified in this study that macrophages are programmed to pyroptosis and apoptosis in response to ZIKV infection, but whether or not PANoptosis occurred remains to be further examined.

In ZIKV-infected macrophages, since nonstructural protein NS5 is required for NLRP3 activation [16], the pyroptosis is very likely to be triggered by NS5. We cannot determine the exact mechanism about how apoptosis is induced but very likely the induction of TNF-α [45] may not play an important factor in activating the cascade of caspases leading to the processing of pro-caspase-3. We have no clue at this stage whether these programed cell death pathways may regulate each other in ZIKV-infected macrophages. Even though caspase-3 can cleave GSDMD, leading to pyroptosis [46], we showed in this study that ZIKV-triggered pyroptosis in macrophages was caspase-1 dependent via a canonical approach. Concomitant occurrence of more than one cell death pathways, including PANoptosis, may minimize the ability of viruses to overcome cell death signaling and benefit the host to contain and clear viral infection. Our findings in this report may help us further understand the complicated mechanisms how macrophages interact with ZIKV and viral pathogenesis in infected humans.

## Supporting information

S1 Fig Differentiation and polarization of THP-1 with PMA treatment and ZIKV infection. ZIKV infection induced THP-1 and RAW264.7 cell death. THP-1 cells were pre-treated with or without PMA and later infected with ZIKV at an MOI of 0.1 for 48 hrs. Relative Transcription levels of cell surface markers CD14 (**A**), CD71, CD163, and HLA-DRA (**B**) in THP-1 monocytes were measured with SYBR Green realtime PCR. (**C**) Release of lactate dehydrogenase (LDH) from ZIKV-infected THP-1 cells. PMA-differentiated THP-1 cells were infected with ZIKV (0.1 MOI) and the culture medium was detected for the levels of LDH at various time points p.i. The assay was carried out twice and data were shown as mean+SD, analyzed by unpaired Students t-test. *, P<0.05; **, P<0.01; ***, P<0.001. ns, no significance.
(TIF)

S2 Fig Morphology of PMA differentiated THP-1 and RAW264.7 macrophages infected with ZIKV. Extensive cell damage was observed, including swelling of cell bodies, rupture of cell membranes, and break up into debris of cell bodies in both cultures at 12 and 48 hrs p.i. under a light microscope (Magnification x200).
(TIF)

S1 Table The sequences of the primers for realtime PCR used in this study. (DOCX)

S1 File Western blot raw_images. (PDF)

S1 Raw images (PDF)
