# Supplementary figures and images for: Concomitant pyroptotic and apoptotic cell death triggered in macrophages infected by Zika virus
Source: PLoS One. 2022 Apr 21;17(4):e0257408. doi: 10.1371/journal.pone.0257408 (PMC9022797; doi:10.1371/journal.pone.0257408)

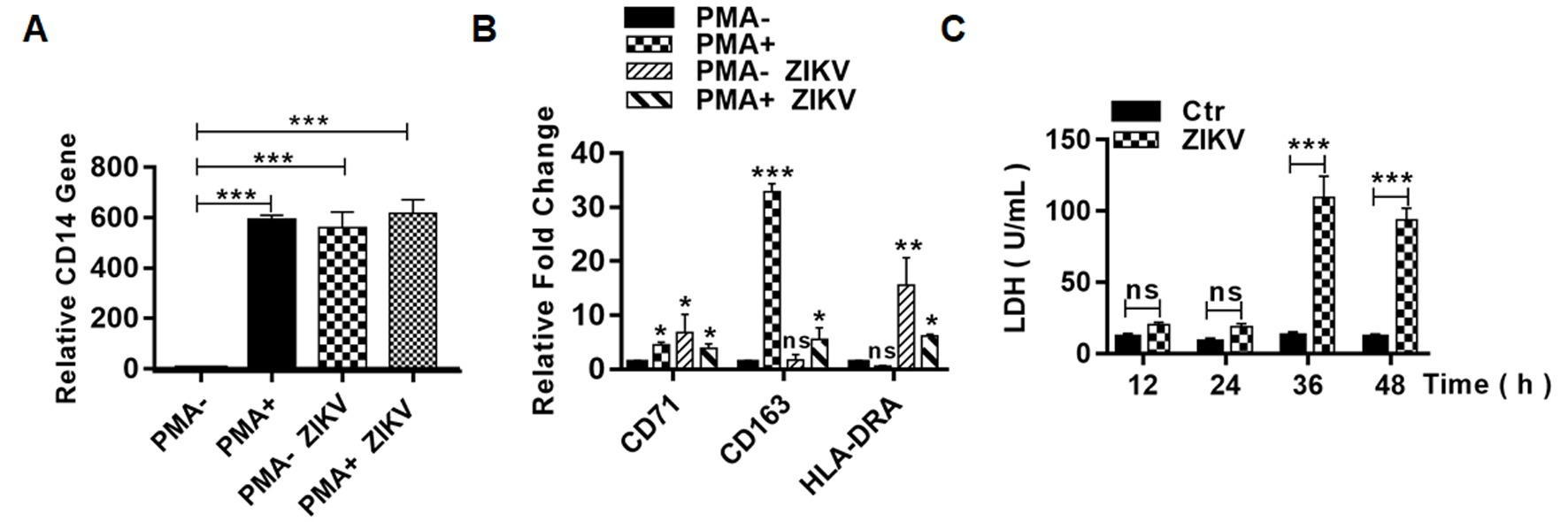

Supplement: S1 Fig — ZIKV infection induced THP-1 and RAW264.7 cell death. THP-1 cells were pre-treated with or without PMA and later infected with ZIKV at an MOI of 0.1 for 48 hrs. Relative Transcription levels of cell surface markers CD14 (A), CD71, CD163, and HLA-DRA (B) in THP-1 monocytes were measured with SYBR Green realtime PCR. (C) Release of lactate dehydrogenase (LDH) from ZIKV-infected THP-1 cells. PMA-differentiated THP-1 cells were infected with ZIKV (0.1 MOI) and the culture medium was detected for the levels of LDH at various time points p.i. The assay was carried out twice and data were shown as mean+SD, analyzed by unpaired Students t-test. *, P<0.05; **, P<0.01; ***, P<0.001. ns, no significance. (TIF) [file pone.0257408.s001.tif]

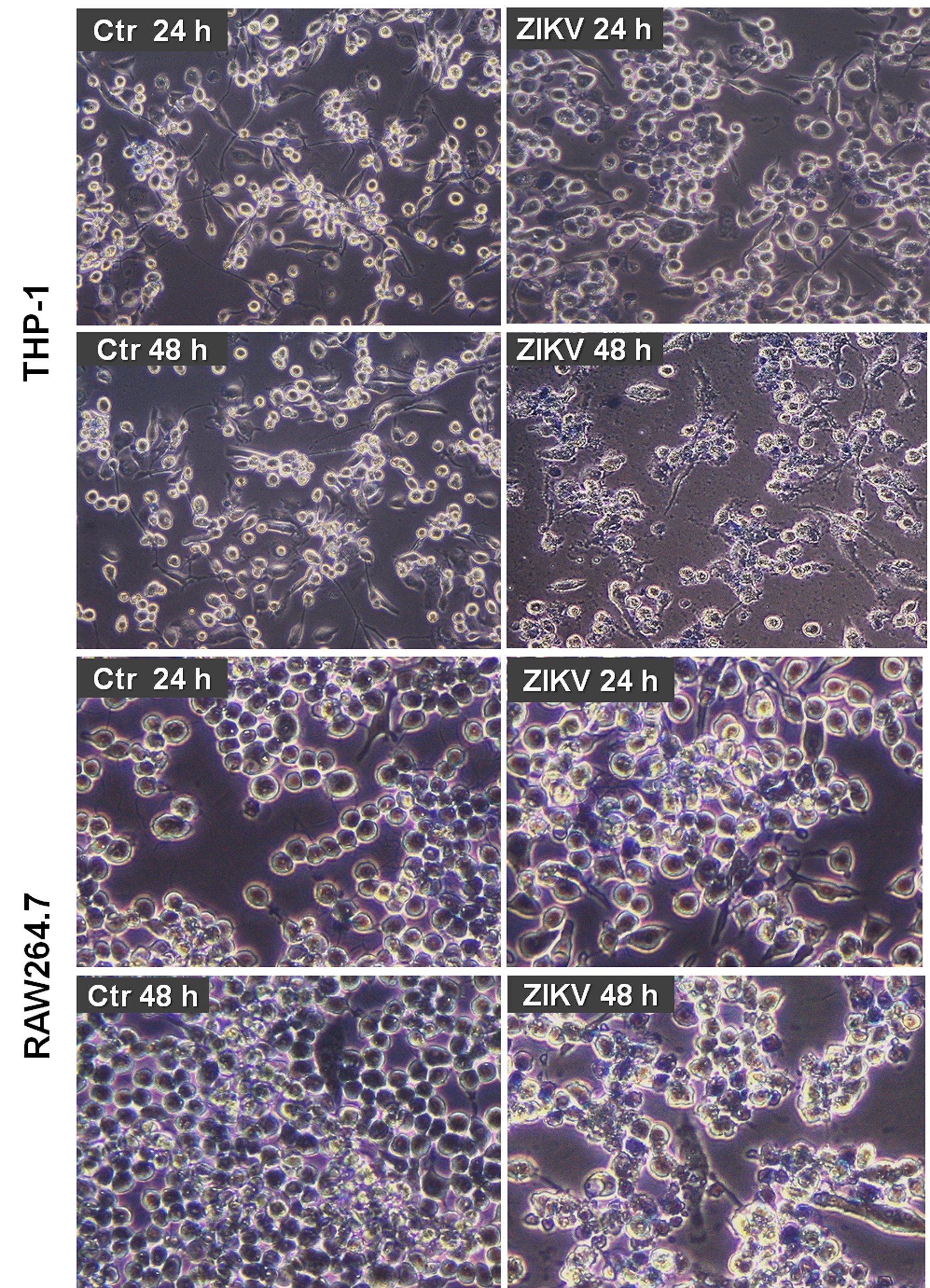

Supplement: S2 Fig — Extensive cell damage was observed, including swelling of cell bodies, rupture of cell membranes, and break up into debris of cell bodies in both cultures at 12 and 48 hrs p.i. under a light microscope (Magnification x200). (TIF) [file pone.0257408.s002.tif]

Figure 1G

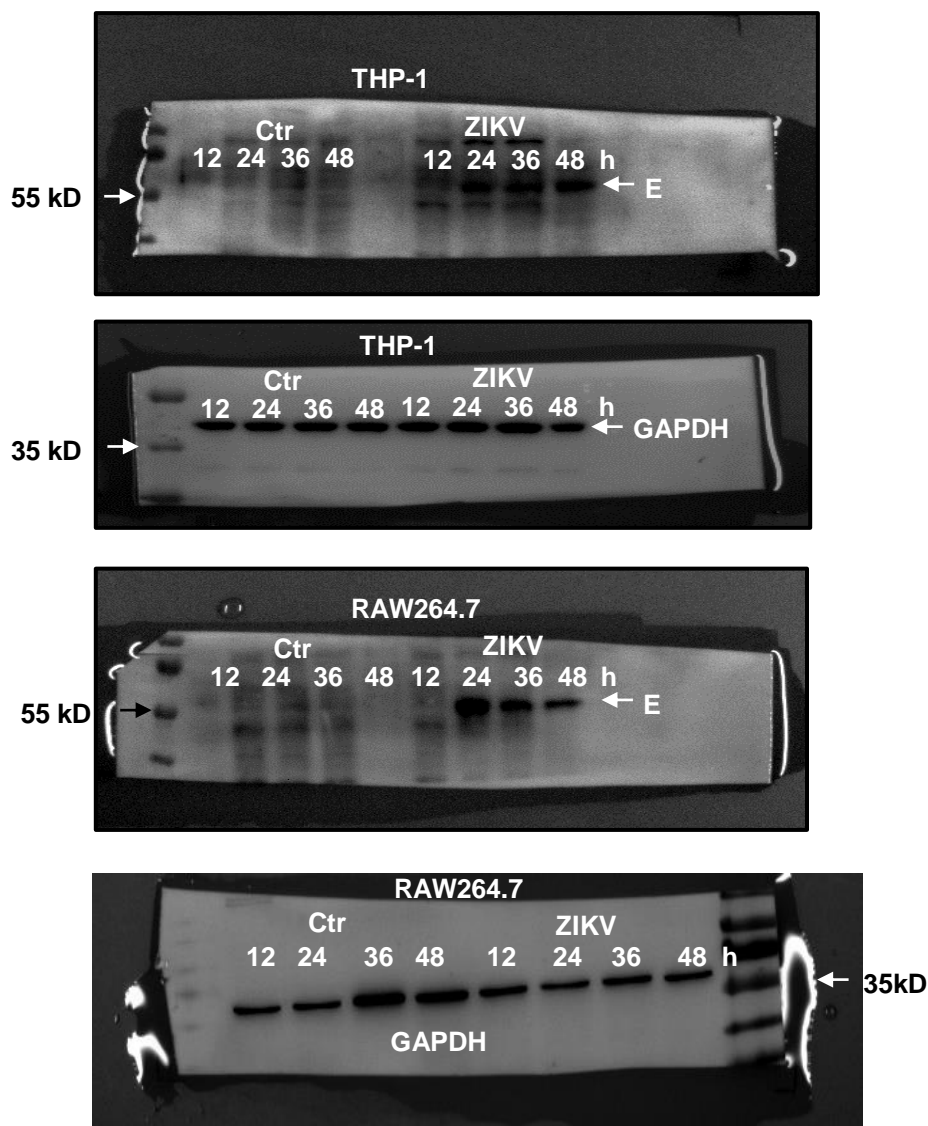

## Figure 3C

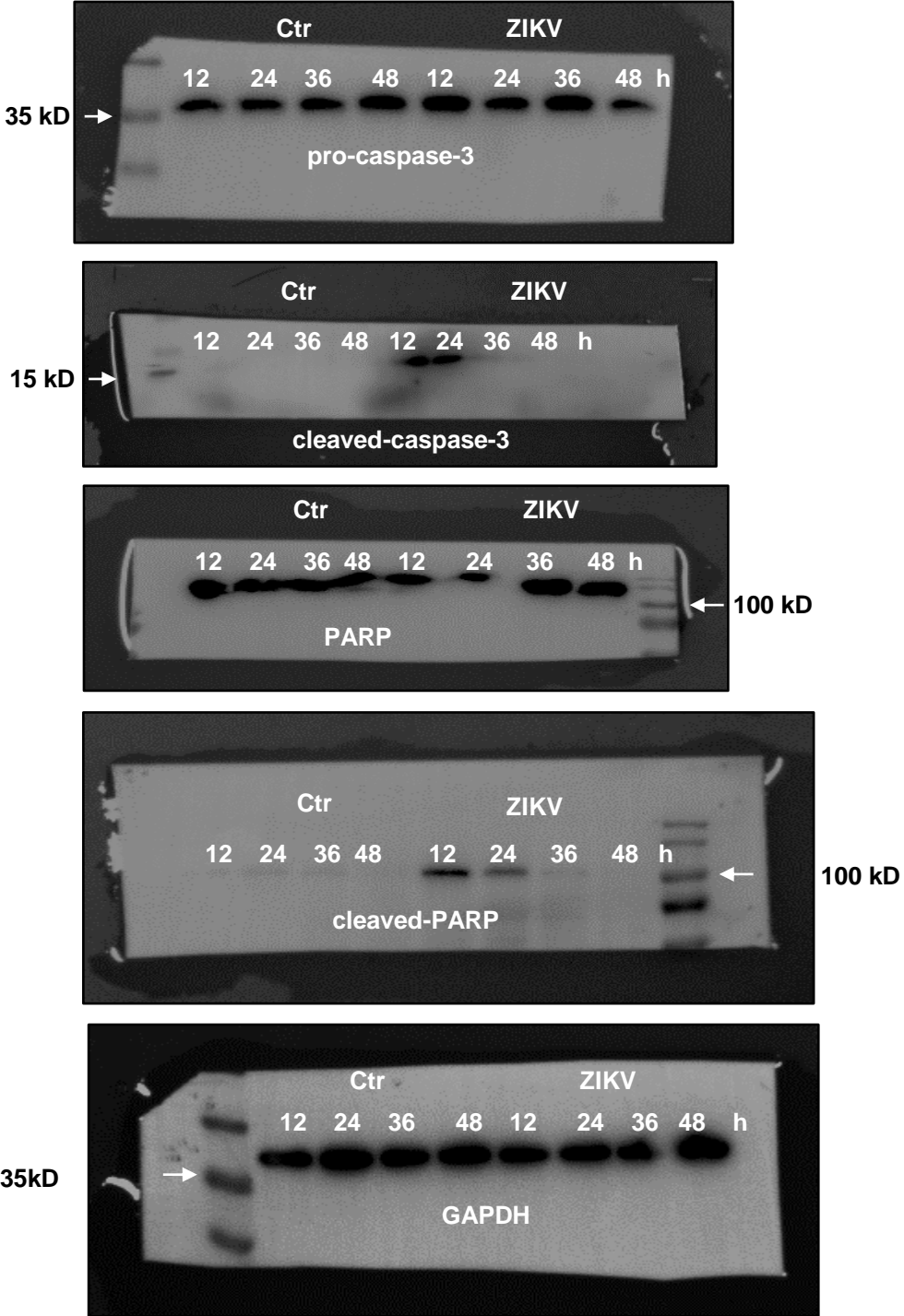

Figure 3D

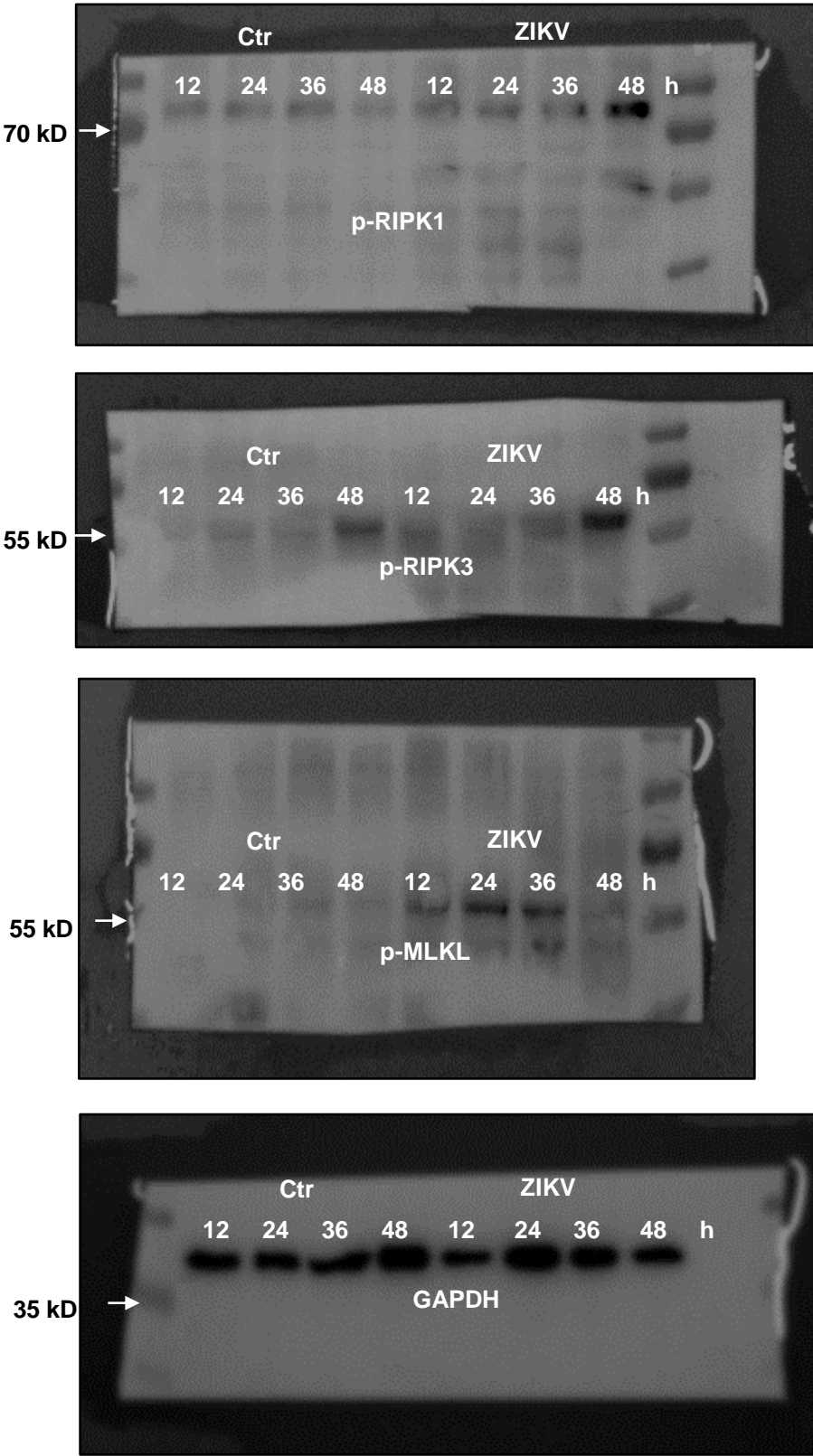

Figure 6A

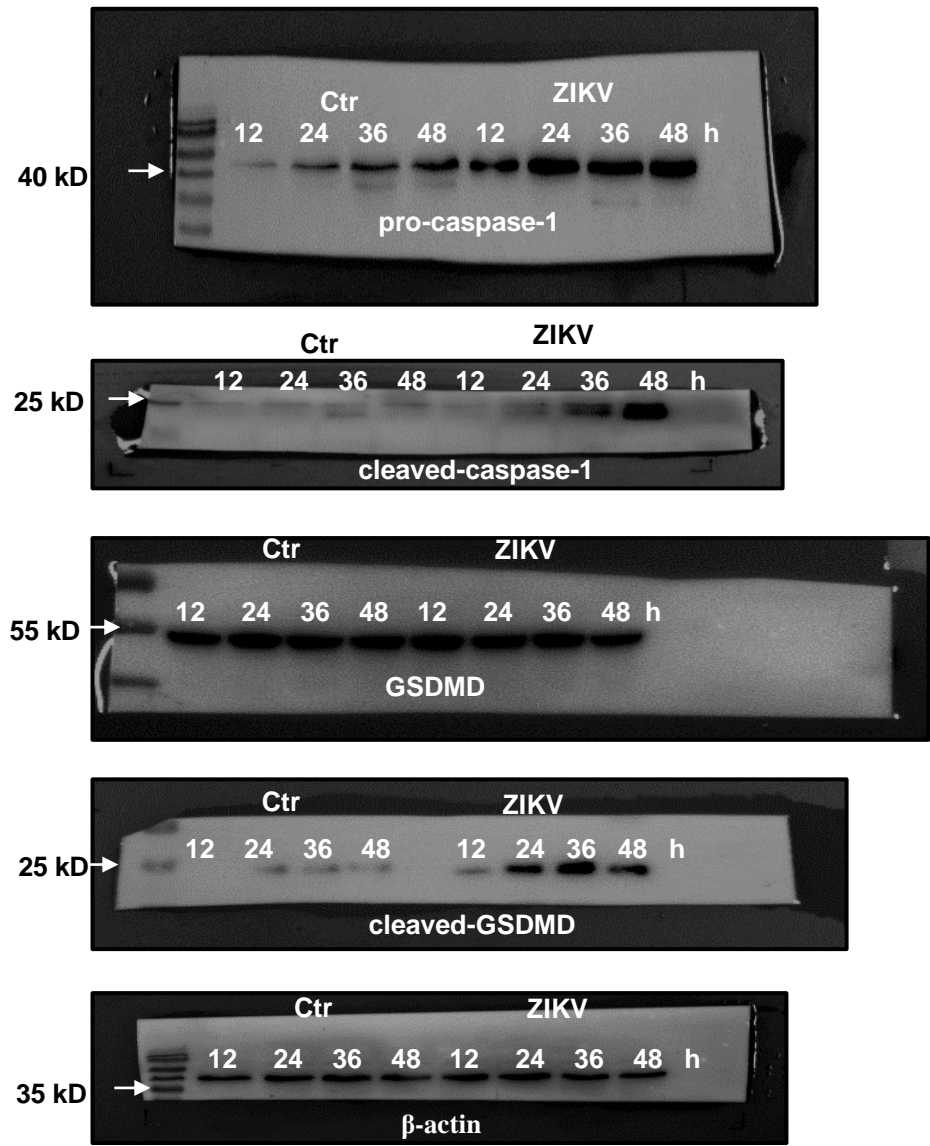

Figure 6B

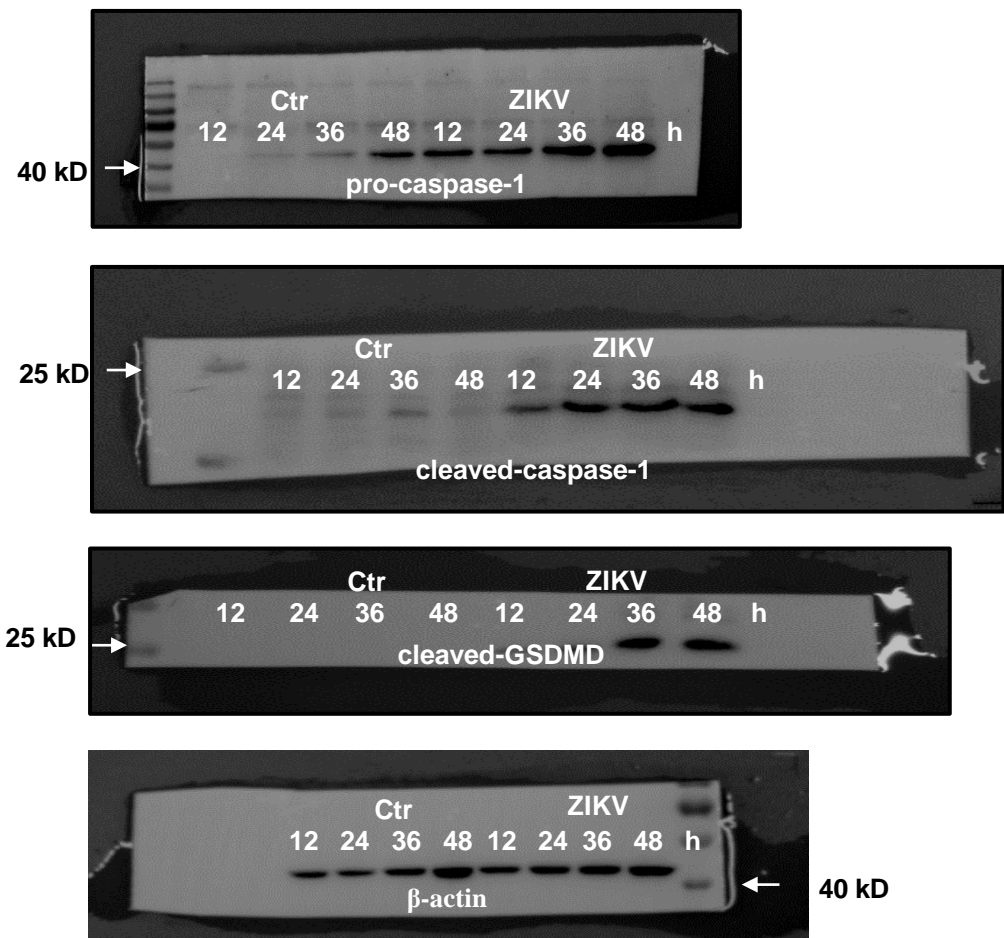

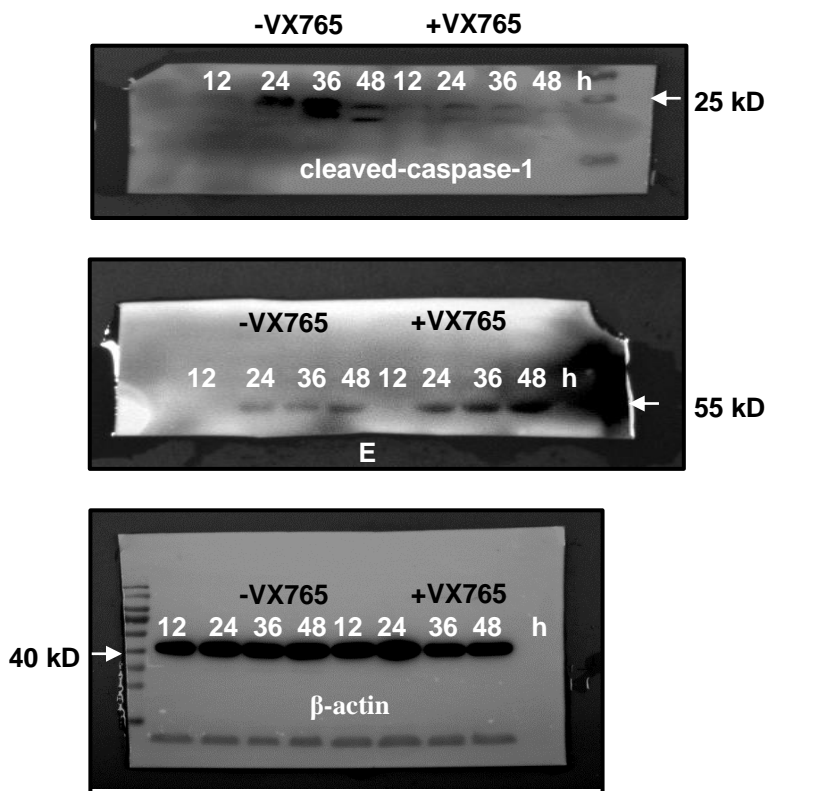

Figure 9A

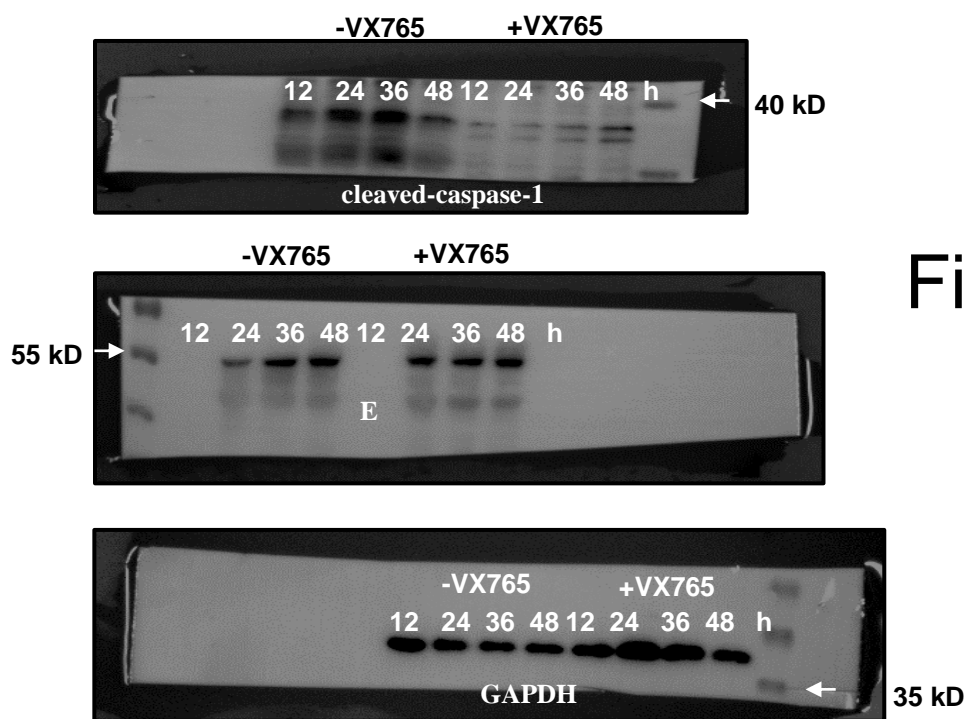

Figure 9B

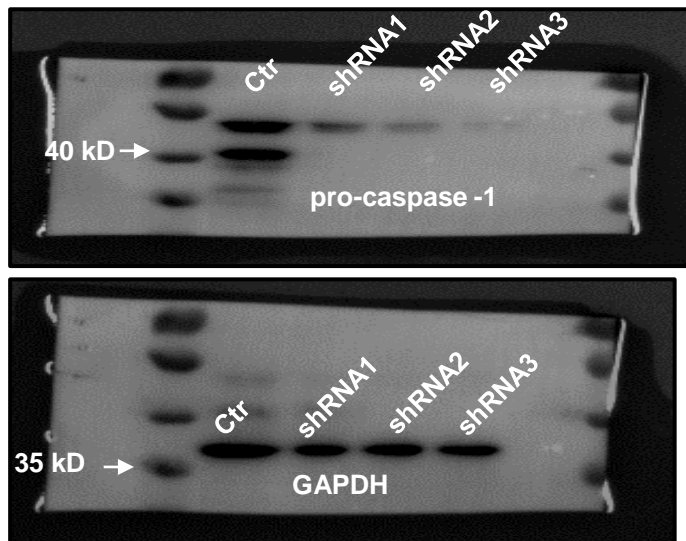

Figure 10A

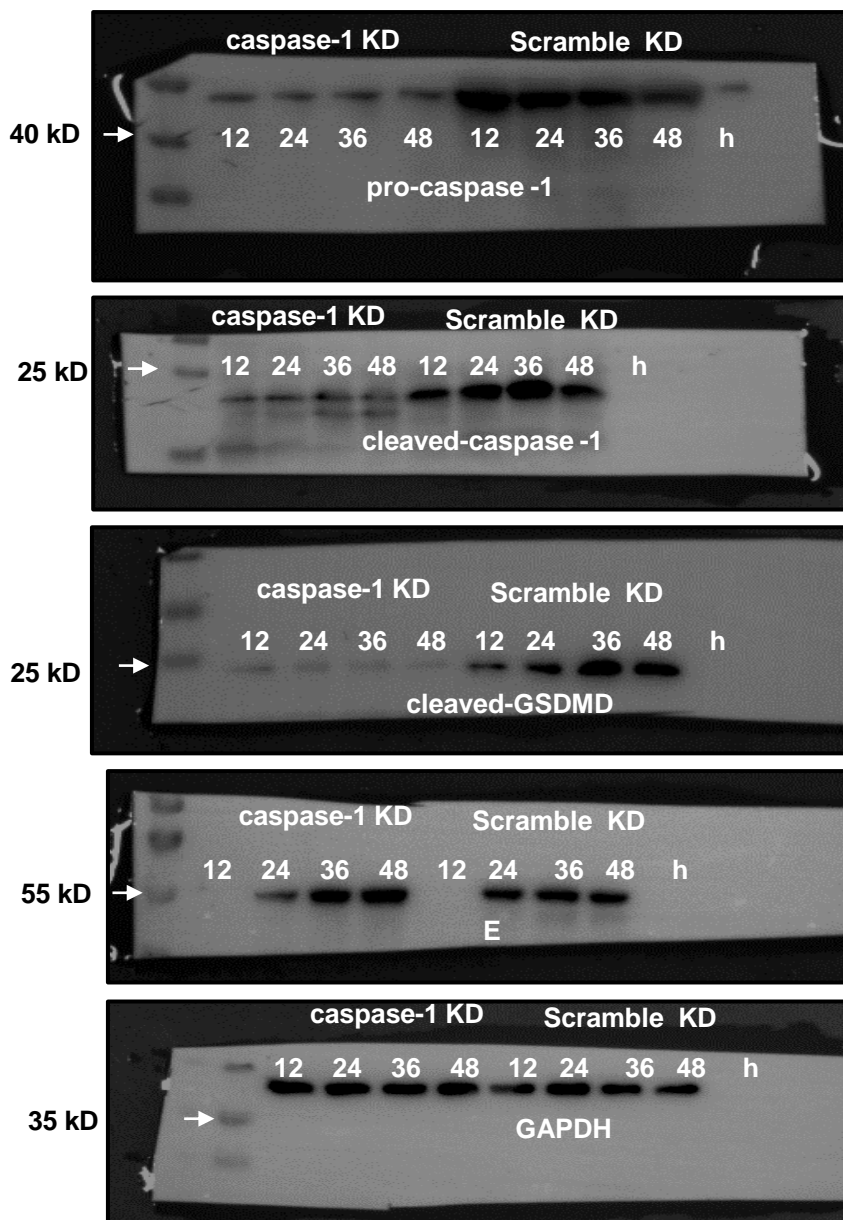

Figure 10B

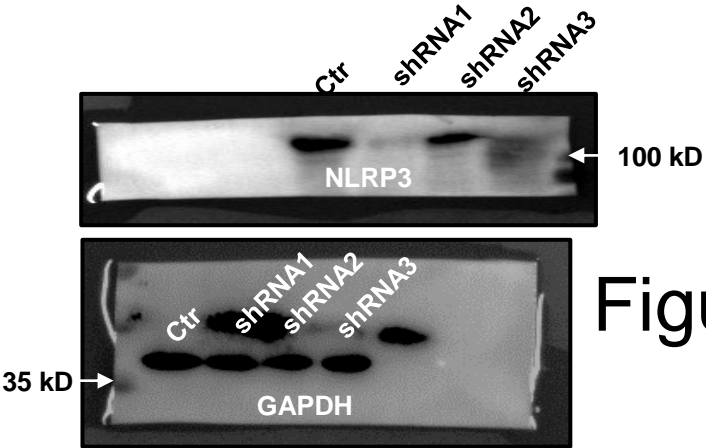

Figure 11A

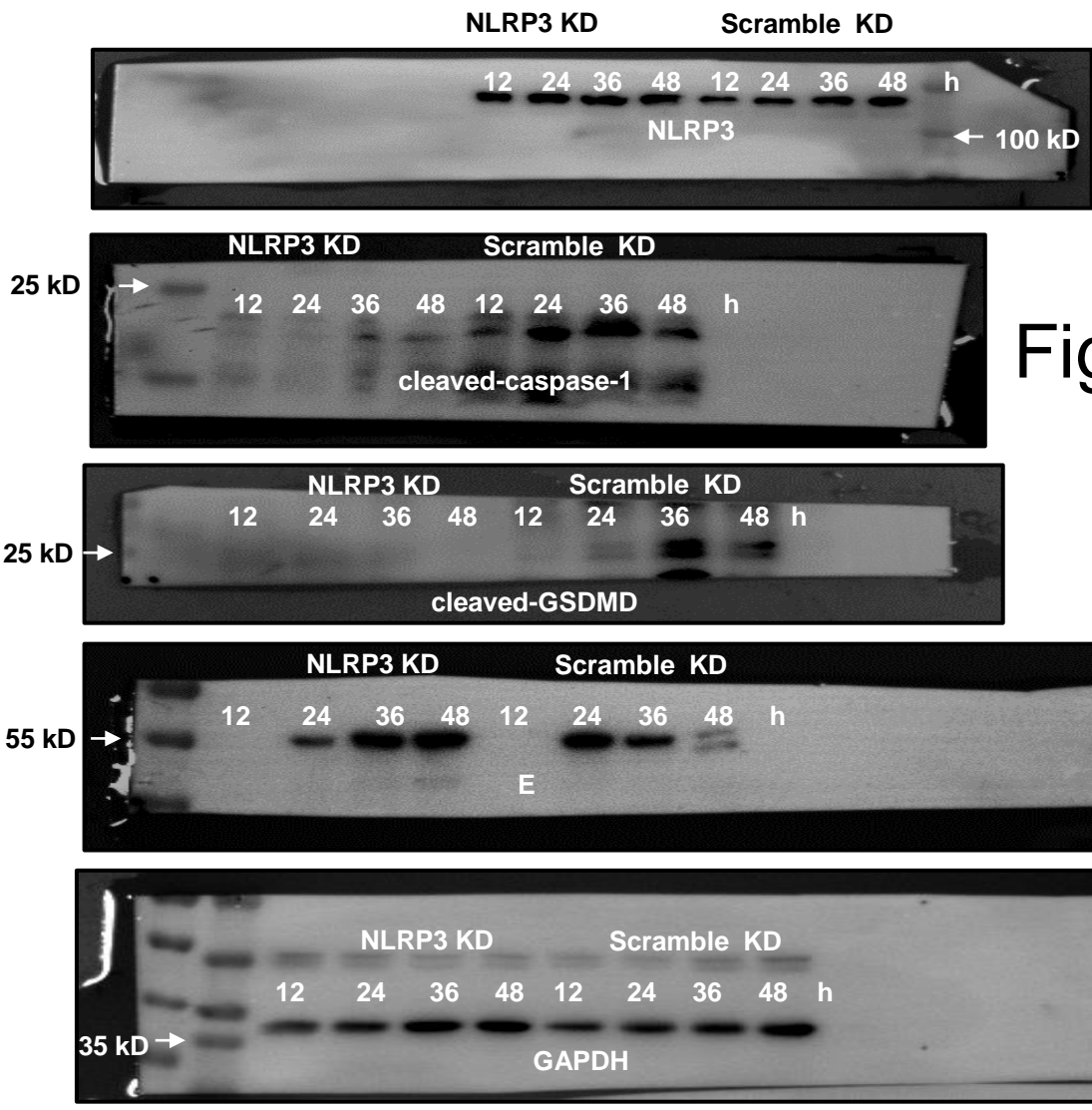

Figure 11B

Supplement: S1 File — (PDF) [file pone.0257408.s004.pdf]
