# Supplementary material for: Concomitant pyroptotic and apoptotic cell death triggered in macrophages infected by Zika virus
Source: PLoS One. 2022 Apr 21;17(4):e0257408. doi: 10.1371/journal.pone.0257408 (PMC9022797; doi:10.1371/journal.pone.0257408)
Supplement: S1 Table — (DOCX) [file pone.0257408.s003.docx]

| Table 1 | Primers used for quantitative PCR analysis. | |  |
| --- | --- | --- | --- |
| Genes | **Forward primer (5′–3′)** | **Reverse primer (5′–3′)** | |
| human-NLRP3 | GATCTTCGCTGCGATCAACA | GGGATTCGAAACACGTGCATTA | |
| human-caspase-1 | GCCTGTTCCTGTGATGTGGAG | TGCCCACAGACATTCATACAGTTTC | |
| human-IL-1β | CCAGGGACAGGATATGGAGCA | TTCAACACGCAGGACAGGTACAG | |
| human-IL-18 | AAAGATAGCCAGCCTAGAGGTA | TGTTATCAGGAGGATTCATTTC | |
| mouse-NLRP3 | TCACAACTCGCCCAAGGAGGAA | AAGAGACCACGGCAGAAGCTAG | |
| mouse-caspase-1 | AACAGAACAAAGAAGATGGCACA | AGCTCCAACCCTCGGAGAAA | |
| mouse-IL-1β | ACTGTTTCTAATGCCTTCCC | ATGGTTTCTTGTGACCCTGA | |
| mouse-IL-18 | GAAAGCCGCCTCAAACCTTC | GGTTGTACAGTGAAGTCGGC | |
| mouse-β-actin | GGTGTGATGGTGGGAATGG | GCCCTCGTCACCCACATAGGA | |
| human-GAPDH | TGCACCACCAACTGCTTAGC | GGCATGGACTGTGGTCATGAG | |
| ZIKV-E | GCCACTTGAAATGTCGCCTGAA | AAGTCCGCTGTAAAGTTCACCG | |
| ZIKV-E-Taq | GGTCAGCGTCCTCTCTAATAAACG | GCACCCTAGTGTCCACTTTTTCC | |
| ZIKV-E-Probe 5’-6-FAM AGCCATGACCGACACCACACCGT -BHQ1-3’ | | | |
